# P selectin promotes SARS-CoV-2 interactions with platelets and the endothelium

**DOI:** 10.1172/JCI184514

**Published:** 2025-11-17

**Authors:** Cesar L. Moreno, Fernanda V.S. Castanheira, Alberto Ospina Stella, Felicity Chung, Anupriya Aggarwal, Alexander J. Cole, Lipin Loo, Alexander Dupuy, Yvonne X. Kong, Lejla Hagimola, Jemma Fenwick, Paul R. Coleman, Rebecca Carr, Tian Y. Du, Tim Ison, Michelle Newton, Maxwell P. Bui-Marinos, Scott B. Cohen, Jennifer A. Corcoran, Daniel Hesselson, Jennifer R. Gamble, Freda H. Passam, Stuart G. Turville, Paul Kubes, G. Gregory Neely

**Affiliations:** 1 Dr. John and Anne Chong Lab for Functional Genomics, Charles Perkins Centre and School of Life and Environmental Sciences, The University of Sydney, Sydney, New South Wales, Australia.; 2 Kirby Institute, University of New South Wales, Sydney, New South Wales, Australia.; 3 Snyder Institute for Chronic Diseases, University of Calgary, Calgary, Alberta, Canada.; 4 Centenary Institute and Faculty of Medicine and Health,; 5 Haematology Research Lab, Heart Research Institute, and; 6 Central Clinical School, Faculty of Medicine and Health, The University of Sydney, Sydney, New South Wales, Australia.; 7 Cell Biology Unit, Children’s Medical Research Institute, Faculty of Medicine and Health, The University of Sydney, Westmead, New South Wales, Australia.; 8 Vascular Biology Program Centenary Institute, The University of Sydney, Sydney, New South Wales, Australia.

**Keywords:** COVID-19, Infectious disease, Virology, Coagulation, Endothelial cells, Platelets

## Abstract

The physiology of SARS-CoV-2 virus/host interactions is not well understood. To better understand host/virus interactions, we performed a CRISPR activation screen to identify host genes that confer resistance to authentic SARS-CoV-2. This highlighted 34 new candidate genes that may alter the course of infection. We validated that 7 of these genes can suppress authentic SARS-CoV-2 infection, including the innate immune receptor P selectin, which increases SARS-CoV-2 spike-dependent binding to cells, while protecting from infection. P selectin also promotes binding to SARS-CoV-2 variants, SARS-CoV-1, and Middle East respiratory syndrome spike proteins, suggesting a general role for P selectin in highly pathogenic coronavirus infections. Importantly, P selectin protein expression driven by synthetic mRNA can block SARS-CoV-2 infection. Naturally, P selectin is expressed on platelets, and we show that it promotes spike-mediated platelet aggregation. P selectin is also expressed on the endothelium, where SARS-CoV-2 spike interactions are also P selectin dependent. In vivo, SARS-CoV-2 uses P selectin to home to capillary beds where the virus interacts with platelets and endothelium, and blocking this interaction can clear vascular-associated pulmonary SARS-CoV-2.

## Abstract 2

This study identifies new gene regulators for cellular SARS-CoV-2 infection. Of these, we describe new roles for the protein P-selectin. This protein when expressed in platelets and the vasculature is able to bind virus, and it is required for Spike-mediated aggregation, but it does not lead to infection in cellular models. Conversely, when P-selectin is blocked infection outcomes worsen in animal models.

## Graphical abstract

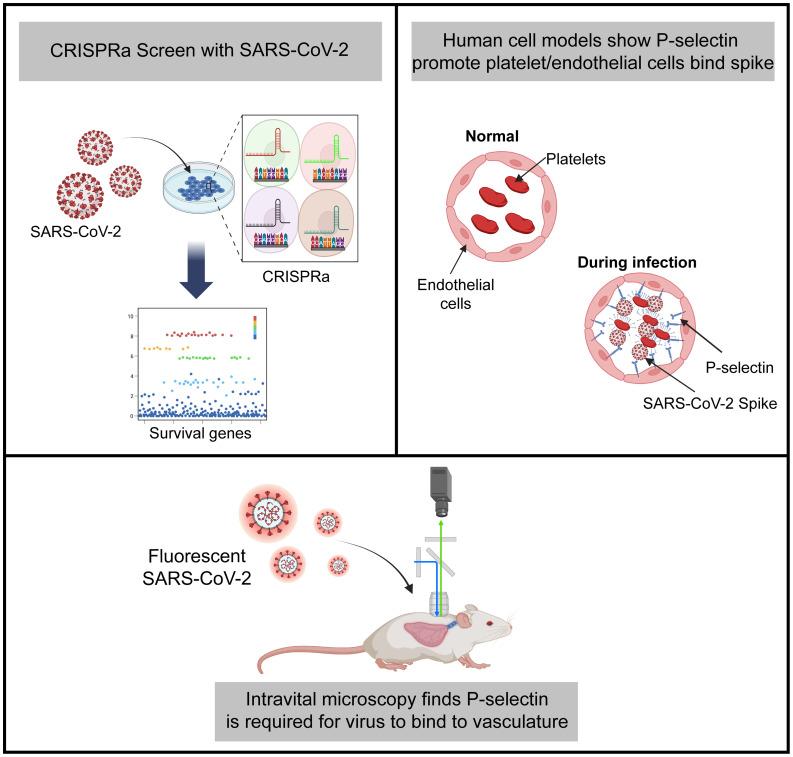

## Introduction

The emergence of SARS-CoV-2, the virus responsible for COVID-19, has resulted in a prolonged global health challenge. Between 2020 and 2021, an estimated 14.83 million deaths were attributed to the pandemic, underscoring its devastating impact (1). Although widespread vaccination has markedly reduced disease severity and mortality, new variants capable of evading the immune response continue to spread at high frequency (2), and vaccination rates are decreasing globally (3). SARS-CoV-2 infection can cause a range of symptoms, from mild to severe. The main cause of death from COVID-19 is respiratory failure (4), but other common complications include multi-organ failure (5), as well as thrombotic events (6). To understand COVID-19 disease, and to develop more effective therapies, we require a fundamental molecular insight of how SARS-CoV-2 can interact with the host.

## Results

### Host factors sufficient for resistance to authentic SARS-CoV-2.

**Figure 1:**
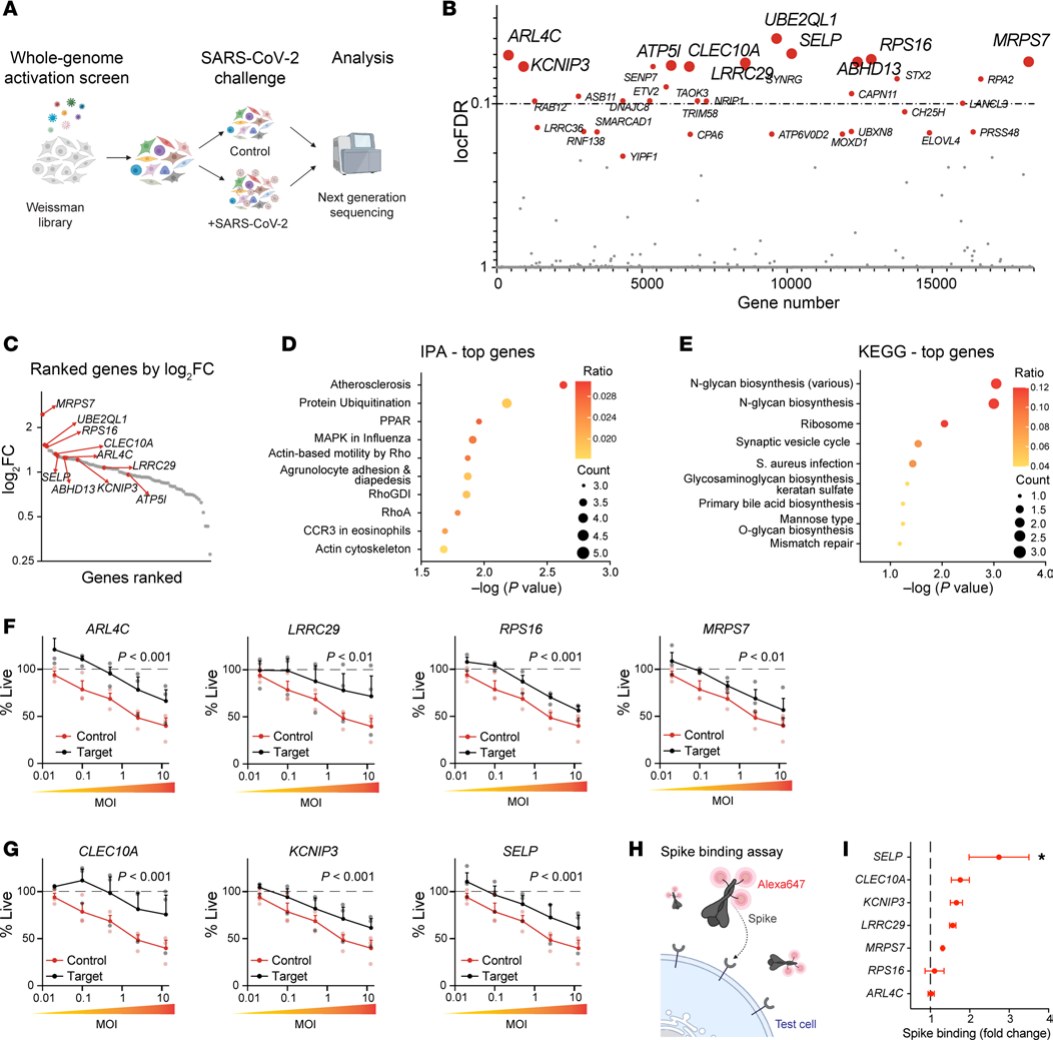
CRISPRa screen against authentic SARS-CoV-2. (**A**) Schematic showing whole-genome CRISPRa screen in HEK293*-ACE2* cells using the Weissman library (https://weissman.wi.mit.edu/crispr/). Cells were transduced with lentiviral pools encoding individual activation sgRNAs tiling the genome. Cells were then inoculated with authentic SARS-CoV-2 or parallel controls, and guides promoting SARS-CoV-2 were identified by sequencing. (*N* = 2.) (**B**) Top genes by local FDR (locFDR) (<0.4 considered significant) identified after selection. See also Supplemental Table 1. (**C**) Plot showing sgRNA enrichment (log fold change) versus gene ranking. (**D** and **E**) Top pathways identified using Ingenuity Pathway Analysis (IPA) (**D**) or KEGG pathways (**E**). (**F** and **G**) Independent validation of top hits including cytosolic (*ARL4C*, *LRRC29*, *RPS16*) or mitochondrial proteins (MRPS7) (**F**), and membrane proteins promoting SARS-CoV-2 resistance (48 hours after inoculation) (**G**). Significance was determined by 2-way ANOVA and Dunnett’s test. (*N* = 3.) (**H**) Diagram of SARS-CoV-2 FACS spike binding assay. (**I**) CRISPRa-driven expression of SELP/P selectin promotes binding to SARS-CoV-2 spike protein. Significance was obtained by 1-way ANOVA and Šidák’s test, *P < 0.05. (*N* = 3.)

To identify human genes that can modify SARS-CoV-2 infection, we used a whole-genome CRISPR activation (CRISPR-SunTag system) strategy (Figure 1A). Cells (HEK293-*ACE2*) were transduced with a CRISPR activation (CRISPRa) sgRNA lentivirus library and then infected with a SARS-CoV-2 A.2.2 clinical isolate at a 0.02 MOI leading to death in 90% of cells after 3 days. Surviving cells were recovered and lysed before integrated sgRNA sequences were amplified by PCR and sequenced. The ratio of each sgRNA in control versus SARS-CoV-2–infected cells was determined, and overrepresented guides, i.e., guides that may confer resistance to SARS-CoV-2, were identified using a mixed hierarchical statistical model optimized for whole-genome CRISPR activation/inhibition screen (7). In this analysis, guide distribution is evaluated compared with negative control sgRNA guide distribution, where deviation from control distribution is indicative of functional selection. Our quality control negative sgRNA counts showed normally distributed curves (Supplemental Figure 1, B and C; supplemental material available online with this article; https://doi.org/10.1172/JCI184514DS1), and using a false discovery rate (FDR) less than 0.1 as a cutoff, we identified 34 genes that conferred some protection to SARS-CoV-2 infection (Figure 1, B and C, and Supplemental Table 1). Conversely, we did not detect statistically significant sgRNA promoting sensitivity to live SARS-CoV-2 (Supplemental Table 2). Bioinformatic analysis of the top 100 sgRNA-targeted genes identified significant enrichments in canonical pathways involved in atherosclerosis, ubiquitination, and influenza infection (Figure 1D). Additional characterization of our top resistance genes using the Kyoto Encyclopedia of Genes and Genomes (KEGG) highlighted that the main molecular processes leading to protection involve protein glycosylation (Figure 1E).

The top 10 SARS-CoV-2 resistance genes were evaluated using 2 sgRNAs per candidate. We confirmed that 7 of these targets conferred resistance to infection, while 3 did not (Supplemental Figure 1D). Identified resistance genes included cytosolic and ribosomal proteins (Figure 1F), as well as mitochondrial proteins or cell surface proteins (Figure 1G). For example, we identified *ARL4C*, encoding a member of the ARF family of GTPases, which participates in various cellular processes, including vesicle trafficking and cholesterol export (8); *LRRC29* (FBXL9P), which is a component of the SKP1–cullin–F-box complex that regulates phosphorylation-dependent ubiquitination (9); *RPS16*, which encodes a component of the 40s cytoplasmic ribosome (10); and *MRPS7*, which encodes a mitochondrial ribosomal 28S subunit protein (11). Of the top-ranked SARS-CoV-2 resistance genes encoding membrane-localized proteins, we found *CLEC10A*, coding for a type II transmembrane lectin that is involved in immune response and inflammation, and which has been reported to interact with spike protein (12); *KCNIP3*, a potassium channel–interacting protein (13) that can also act as a Ca^2+^-regulated transcriptional repressor (14); and *SELP*, which encodes the protein P selectin, a critical cell adhesion molecule that also controls fibrin deposition (15–17). We tested whether any of these membrane-localized resistance genes were sufficient to promote SARS-CoV-2 spike binding (Figure 1H), and found that P selectin promoted cellular interactions with spike (Figure 1I).

### P selectin interacts with pathogenic coronavirus spike proteins and protects target cells.

**Figure 2:**
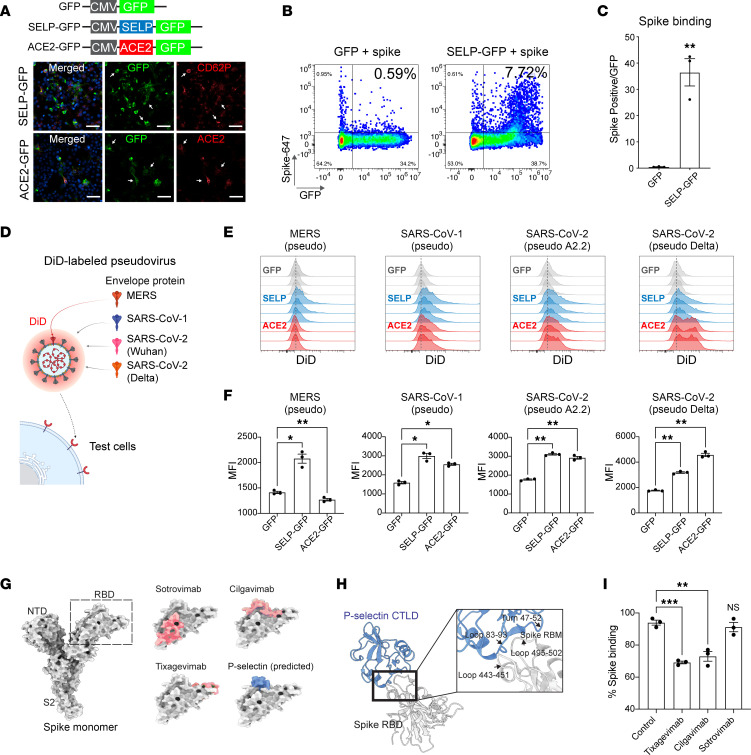
P selectin interaction with pathogenic coronavirus spike proteins. (**A**) Ectopic ACE2 or P selectin expression strategy. Scale bars: 60 µm. (**B**) Representative flow cytometry plots showing binding of P selectin to SARS-CoV-2 spike protein. (**C**) Quantification of **B**. Significance was determined by paired 2-tailed *t* test, **P < 0.01. (*N* = 3.) (**D**) Schematic of DiD-labeled pseudolentivirus assay used in **E** and **F**. (**E** and **F**) Flow cytometry histograms (**E**) and quantification of binding intensity (**F**) presented for pseudovirus expressing MERS, SARS-CoV-1, and SARS-CoV-2 A2.2 and Delta variants. MFI, mean fluorescence intensity. Significance was determined by 1-way ANOVA and Dunnett’s test, *P < 0.05, **P < 0.01. (*N* = 3.) (**G**) Binding sites (red) for blocking antibodies AZD1061 (cilgavimab), AZD8895 (tixagevimab), and S309 (sotrovimab) and predicted binding site for P selectin (blue) on SARS-CoV-2 spike glycoprotein (Protein Data Bank: 7WK2) (64). (**H**) Key predicted interactive regions between P selectin and spike RBD. C-type lectin-like domain, CTLD. (**I**) Binding of antibody-blocked spike protein in HEK293T cells expressing P selectin. Significance was determined by 1-way ANOVA and Dunnett’s test, **P < 0.01, ***P < 0.001. (*N* = 3).

P selectin is expressed on activated platelets and endothelium, where it controls platelet aggregation and leukocyte recruitment to sites of inflammation, especially sites of vascular injury (18, 19). Since inflammation, platelet aggregation, and vascular injury are all hallmarks of COVID-19, we considered the role of P selectin in SARS-CoV-2 biology. To confirm our CRISPRa results, we used cDNA to express GFP control, ACE2, or P selectin (Figure 2A) and then evaluated impact on SARS-CoV-2 spike binding and pseudo– or authentic SARS-CoV-2 infection. Indeed, ectopic expression of P selectin (or ACE2) was sufficient to confer SARS-CoV-2 spike binding (Figure 2B, Supplemental Figure 2A, quantified in Figure 2C). P selectin is a lectin that binds to glycoproteins, and it is possible that P selectin shows broad specificity for other glycosylated coronavirus spike proteins. To test this, we generated individual pseudoviruses expressing spike proteins from highly pathogenic members of the Coronaviridae family. To visualize interactions, we labeled pseudoviruses with the lipophilic fluorescent probe DiD (20) and tested whether expressing P selectin could promote cell/pseudovirus interactions (Figure 2D). As reported (21, 22), ACE2 can bind SARS-CoV-1 and SARS-CoV-2 spike, but not Middle East respiratory syndrome (MERS) spike, whereas P selectin can promote binding to both SARS and MERS spike proteins (Figure 2, E and F). This interaction is spike dependent, since no significant increase in binding was observed when a bald virus was used (Supplemental Figure 1, F and G). Moreover, as described by others (23), ACE2 showed enhanced affinity for SARS-CoV-2 Delta spike, whereas P selectin showed comparable binding to all pathogenic coronavirus spike proteins tested (Figure 2, E and F). To further characterize P selectin/spike binding, we used AlphaFold 3 (24) to predict where P selectin and spike receptor-binding domain (RBD) interact (Figure 2, G and H). We tested this prediction using blocking antibodies mapped to specific regions of the RBD (25, 26) (Figure 2G). We observed reduced spike interactions in P selectin–expressing HEK293 cells when spike protein was preincubated with antibodies targeting topologies near the P selectin/spike predicted binding site. Here, blocking with cilgavimab and tixagevimab, but not with sotrovimab (Figure 2I and Supplemental Figure 2C), significantly reduced spike interactions. Overall, these results show that ectopic P selectin expression increases cellular interactions with multiple coronavirus proteins, and uses SARS-CoV-2 spike RBD motifs for binding.

**Figure 3:**
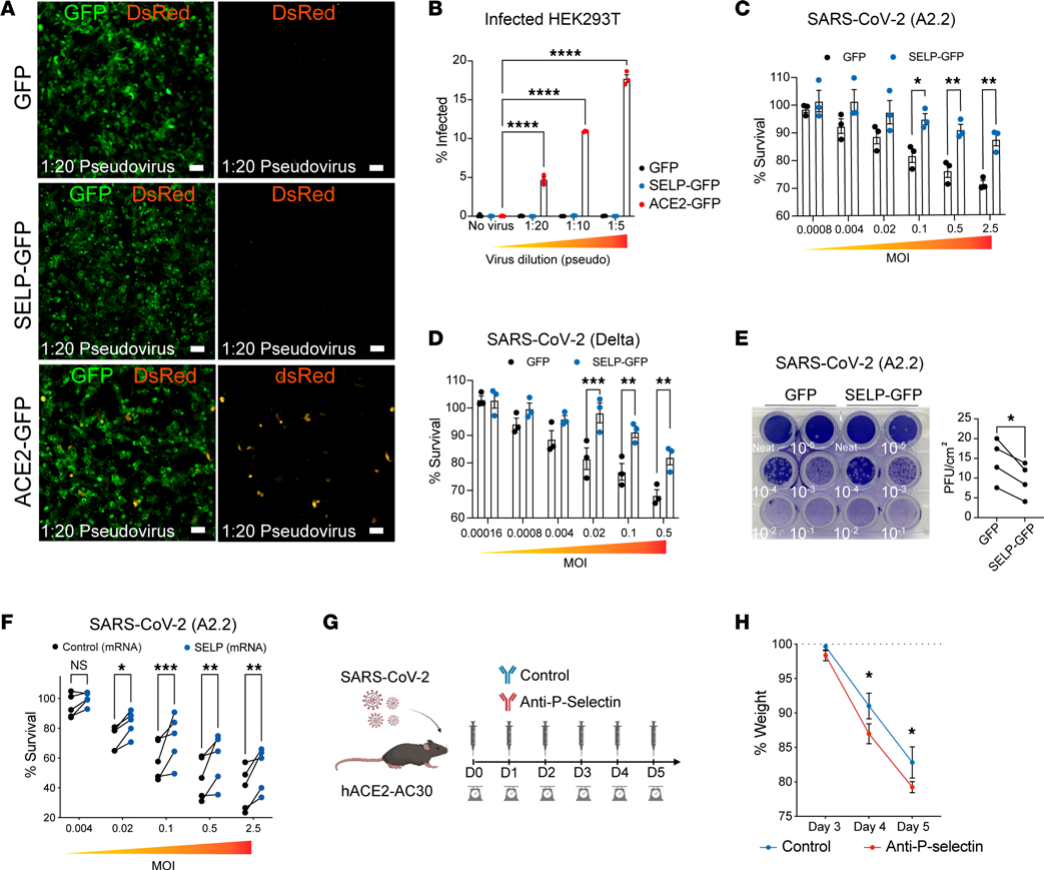
P selectin expression suppresses SARS-CoV-2 infection. (**A**) Images of ACE2- or P selectin–expressing HEK293T cells infected with SARS-CoV-2 spike-expressing pseudolentivirus. Scale bars: 60 μm. (**B**) Quantification of **A**. Significance was determined by 2-way ANOVA and Dunnett’s test, ****P < 0.001. (*N* = 3.) (**C** and **D**) Infection of HEK293-ACE2/TMPRSS2–expressing cells (55) with SARS-CoV-2 A2.2 (**C**) or Delta (**D**) variant is suppressed by coexpression of *SELP* cDNA. Significance was determined by 2-way ANOVA and Šidák’s test. *P < 0.05, **P < 0.01, ***P < 0.001. (*N* = 3). (**E**) Plaque assay showing plaque-forming units (PFU) in VeroE6/TMPRSS2 cells. Significance was determined by paired *t* test, *P < 0.05. (*N* = 4.) (**F**) Infection of HEK293-ACE2/TMPRSS2–expressing cells with SARS-CoV-2 A2.2 is suppressed by *SELP* mRNA expression. Significance was determined by 2-way ANOVA and Šidák’s test, *P < 0.05, **P < 0.01, ***P < 0.001. (*N* = 3.) (**G**) Schematic showing infection of mice with a lethal dose of SARS-CoV-2 and daily injection of P selectin blocking antibody to evaluate infection course. (**H**) Body weight changes after lethal SARS-CoV-2 inoculations in the presence of P selectin blocking antibody. Significance was determined by 2-way ANOVA and Šidák’s test, *P < 0.05. (*N* = 8–9.)

We next assessed whether SARS-CoV-2 can use P selectin as an entry receptor for infection. To this end, we generated a DsRed-SARS-CoV-2 pseudovirus expressing truncated spike protein (27), allowing for dose-dependent quantification of spike-mediated infection. While pseudovirus can infect *ACE2*-expressing cells as expected, P selectin expression was not sufficient to confer SARS-CoV-2 pseudovirus tropism (Figure 3, A, quantified in B).

Authentic SARS-CoV-2 infects cells primarily via an ACE2/TMPRSS2 entry pathway (28). Since P selectin can antagonize both pseudovirus and authentic infection of ACE2-expressing cells, we hypothesized that it would also block infection of ACE2/TMPRSS2^+^ HEK293 target cells. Accordingly, ACE2/TMPRSS2/SELP^+^ cells showed a dose-dependent protection from authentic SARS-CoV-2 infection compared with cells transfected with GFP control vector (Figure 3, C and D, for A.2.2 and Delta variants), while reducing A.2.2 viral copies in the supernatant of inoculated cultures (Supplemental Figure 1H). This protection extended to VeroE6/TMPRSS2 cells transfected with *SELP*, where we observed decreased A.2.2 viral plaque formation (Figure 3E). Similarly, we observed a comparable protection in ACE2/TMPRSS2^+^ HEK293 cells transfected with a therapeutic *SELP* mRNA (Figure 3F). Seeing that P selectin conferred protection at a cellular level, we proceeded to test its function in an animal model (Figure 3G). Transgenic mice expressing human ACE2 (hACE2) under the ubiquitous CAG-AC-70 promoter are susceptible to SARS-CoV-2 infection, showing severe infection and rapidly succumbing to disease (~6 days after infection), likely because of local infection in the lung and brain microcirculation (29). Despite the rapid decline that occurs with this model, we found that chronically blocking P selectin (Figure 3G) throughout infection could still significantly worsen disease progression as measured by a faster body weight loss before death (Figure 3H and Supplemental Figure 3; ~41% enhanced loss by day 4). All together, these results support that P selectin expression can protect against SARS-CoV-2 infection at a cellular level and in vivo.

### P selectin controls SARS-CoV-2 spike binding to platelets and endothelial cells.

**Figure 4:**
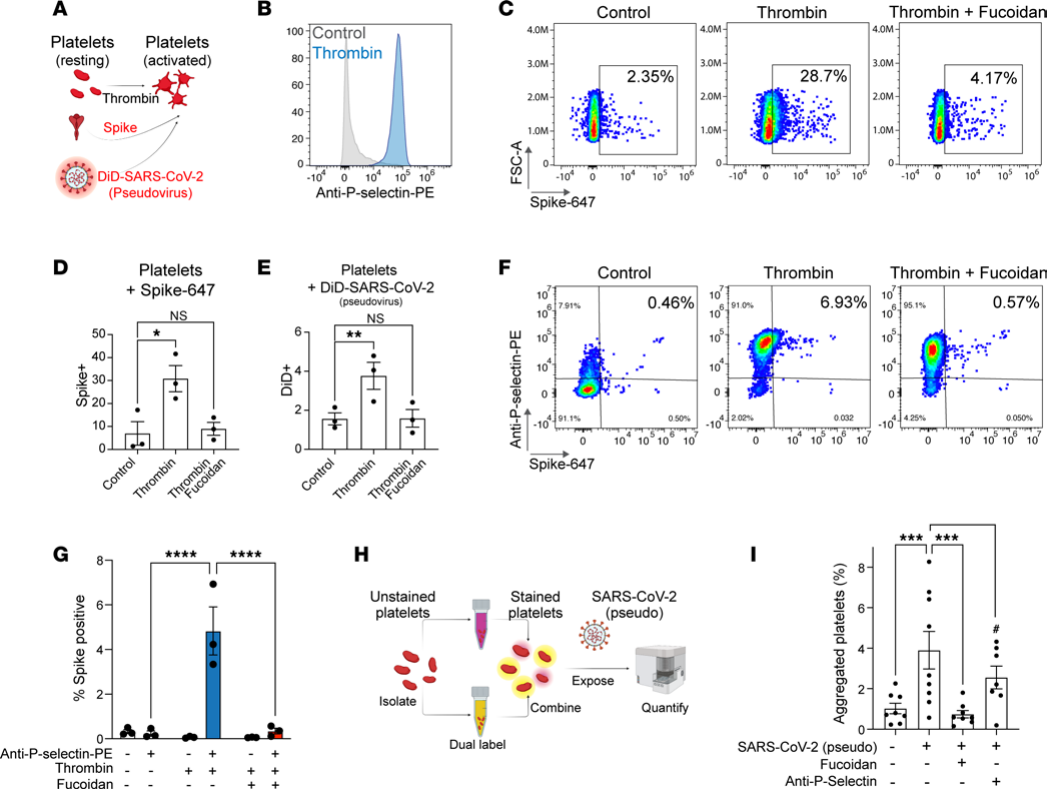
Platelets use P selectin to bind to SARS-CoV-2 spike. (**A**) Schematic for testing P selectin function in platelets. (**B**) Thrombin upregulates cell surface P selectin on platelets. (**C**) Thrombin-activated platelets bind SARS-CoV-2 spike protein, and this is blocked by the P selectin antagonist fucoidan. (**D**) Quantification of **C**. Significance was determined by 1-way ANOVA and Dunnett’s test, *P < 0.05. (*N* = 3.) (**E**) DiD-labeled SARS-CoV-2 pseudovirus binds platelets, and this depends on P selectin. Flow cytometry quantification with significance determined by 1-way ANOVA and Dunnett’s test, **P < 0.01. (*N* = 3.) (**F**) Platelets that bind SARS-CoV-2 spike protein are P selectin positive. (**G**) Quantification of **F**. Significance was determined by 2-way ANOVA and Šidák’s test, ****P < 0.0001. (*N* = 3.) (**H**) Schematic for testing platelet aggregation (33). Isolated platelets were individually labeled with 2 fluorescent probes, and recombined before exposure to SARS-CoV-2 (pseudovirus). Double-positive fractions indicate aggregation. (**I**) Flow cytometry quantification for aggregated platelets. Significance was determined by 1-way ANOVA and Holm-Šidák test, ***P < 0.1; and Holm-Šidák 1-tailed test, ^#^*P* < 0.05. (*N* = 7–9.)

**Figure 5:**
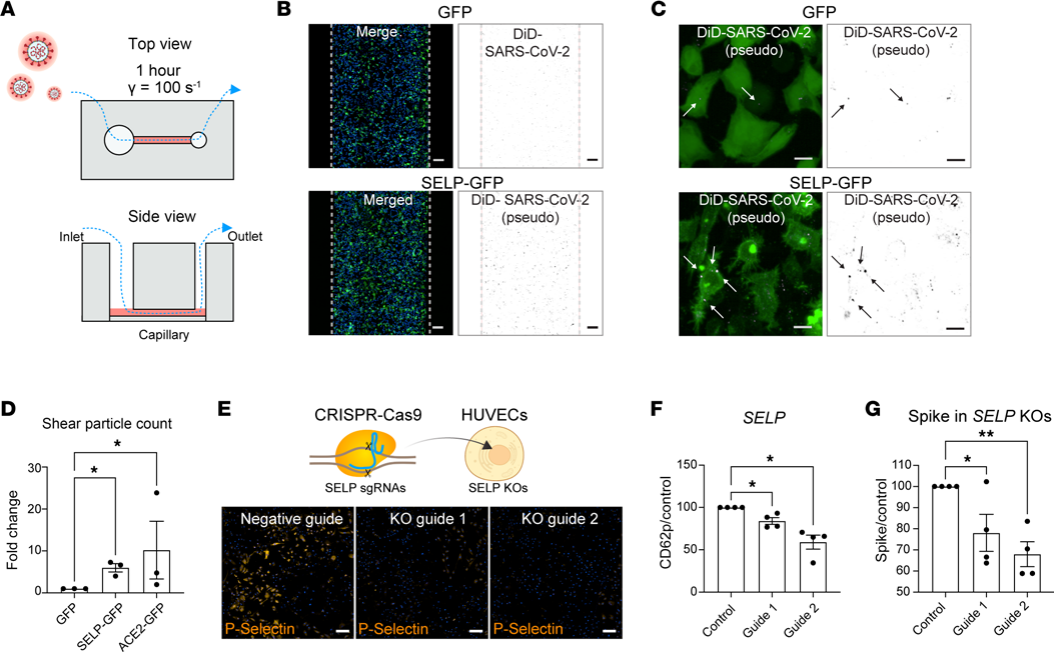
Endothelial cells use P selectin to bind SARS-CoV-2 spike. (**A**) Schematic of microfluidic devices used to study SARS-CoV-2 pseudovirus binding to P selectin under shear conditions. (**B**) Representative images of capillary lined with cells expressing GFP or P selectin–GFP after 1 hour of flow with SARS-CoV-2 pseudovirus–containing medium. Scale bars: 100 μm. (**C**) Representative images detailing accumulation of labeled pseudovirus particles in GFP- or P selectin–GFP–expressing cells. Scale bars: 10 μm. (**D**) Automated quantification of total DiD area/cells within the capillary. Significance was determined by 1-way ANOVA and Dunnett’s test, *P < 0.05. (*N* = 3.) (**E**) CRISPR targeting of *SELP* in primary endothelial cells (HUVECs) reduces P selectin staining (scale bar: 200 μm), quantified by flow cytometry in **F**. Significance was determined by 1-way ANOVA and Dunnett’s test, *P < 0.05. (*N* = 4.) (**G**) CRISPR-targeted SELP-edited HUVECs bind less SARS-CoV-2 spike protein. Significance was determined by 1-way ANOVA and Dunnett’s test, *P < 0.05, **P < 0.01. (*N* = 4.)

In platelets, P selectin is found in α-granules, where it is translocated to the cell surface upon inflammatory or thrombogenic challenges (30) (Figure 4A). Thrombin-activated human primary platelets expressed cell surface P selectin (Figure 4B), and binding to SARS-CoV-2 spike protein was blocked by the P selectin antagonist fucoidan (31) (Figure 4, C, quantified in D). Fucoidan could also block interactions between activated platelets and DiD-labeled SARS-CoV-2 pseudovirus (Figure 4E). Moreover, platelets that bound to SARS-CoV-2 Spike predominantly expressed surface P selectin, where fucoidan blocked this interaction (Figure 4, F, quantified in G). Similarly, we observed that by treatment of activated platelets with a blocking P selectin antibody, binding of spike protein was prevented (Supplemental Figure 3A). COVID-19 increases the risk for platelet aggregation, and this is in part driven by viral protein exposure (e.g., SARS-CoV-2 spike) (32–34). We tested whether incubating platelets with increasing doses of pseudovirus expressing SARS-CoV-2 spike would potentiate P selectin translocation, but we were unable to detect any differences (Supplemental Figure 3B). However, by mixing resting platelets with pseudovirus expressing SARS-CoV-2 spike, we observed spontaneous platelet aggregation (Figure 4H; methodology is adapted from ref. 33), and this effect was reversed completely by the antagonist fucoidan, and partially by anti–P selectin blocking (Figure 4I). Since SARS-CoV-2 spike can interact with P selectin and SARS-CoV-2 virus is found in inflamed tissue and at sites of vascular injury where P selectin is also found (6), we hypothesized that SARS-CoV-2 may use P selectin to control its localization during infection. To this end, we plated GFP or SELP-GFP^+^ cells and then evaluated the ability of P selectin to promote SARS-CoV-2 pseudovirus adhesion under venular flow rates (Figure 5A). Under these conditions, we observed an accumulation of fluorescent SARS-CoV-2 pseudovirus particles in the capillaries plated with cells expressing P selectin–GFP (Figure 5, B–D, and Supplemental Figure 3C) or ACE2 (Supplemental Figure 3D), but not in those plated with GFP control cells. When we quantified the number of foci per cell area, we found a 7-fold increase in SARS-CoV-2 pseudovirus particle adhesion to cells expressing P selectin (Figure 5D), and this increase was also confirmed by mean fluorescence intensity (Supplemental Figure 3C). To determine whether SARS-CoV-2 pseudovirus binding is dependent on native P selectin expression, we targeted *SELP* in primary human umbilical vein endothelial cells (HUVECs) (Figure 5E), observing approximately 20% (guide 1) and 40% (guide 2) reduction of P selectin expression in comparison with a control guide by immunocytochemistry (Figure 5E) and flow cytometry (Figure 5F and Supplemental Figure 3E). Importantly, reducing P selectin expression in HUVECs was sufficient to decrease SARS-CoV-2 spike protein binding to endothelial cells (Figure 5G).

### Anti–P selectin treatment regulates authentic SARS-CoV-2 infection in vivo.

**Figure 6:**
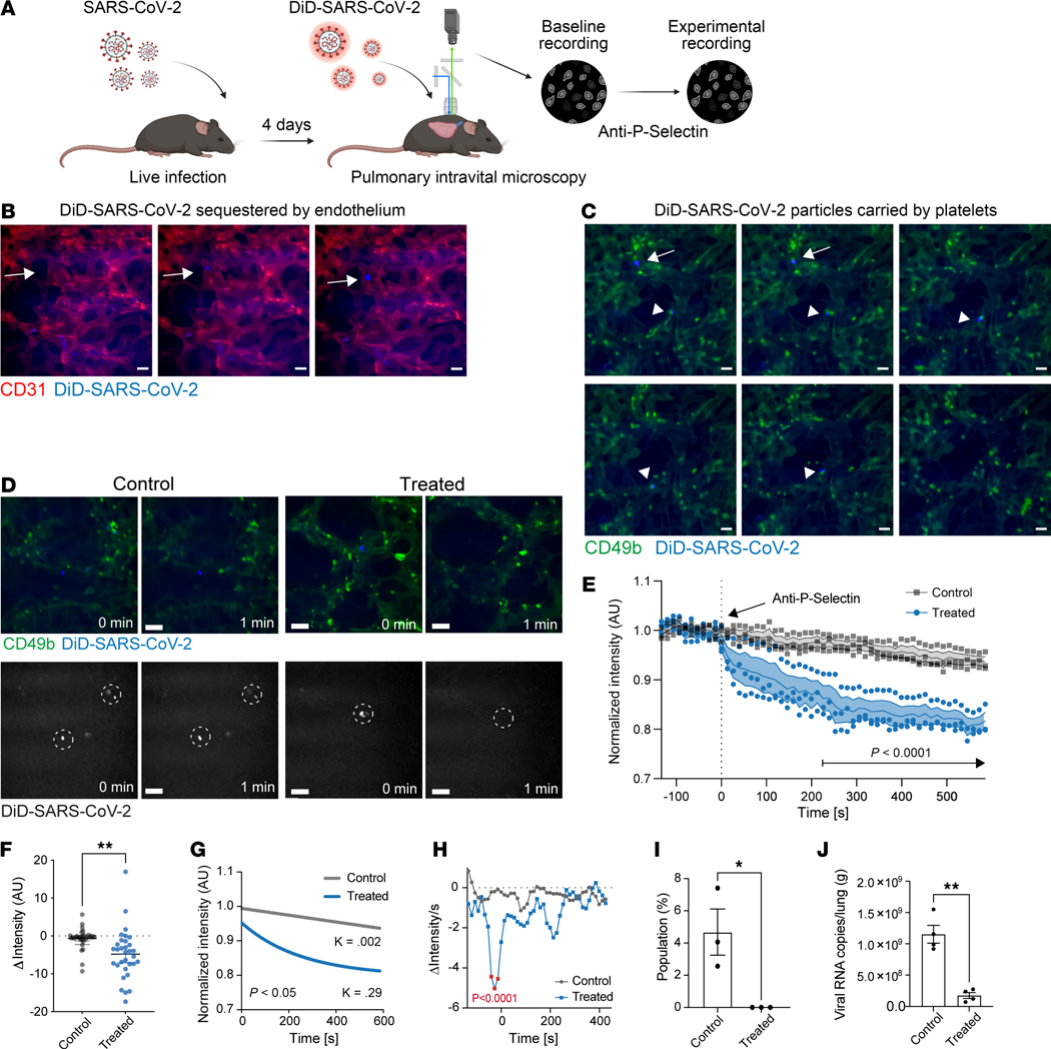
Authentic SARS-CoV-2 uses P selectin to bind to the endothelium in lung capillary beds. (**A**) Schematic of experimental setup. Mice were infected with authentic SARS-CoV-2. Four days later, infected mice were injected i.v. with DiD-labeled authentic SARS-CoV-2, and virus trafficking to the lung was monitored by intravital microscopy. Mice were then treated with anti–P selectin, and SARS-CoV-2 interactions with platelets (anti-CD49b–FITC) and the endothelium vasculature were monitored. (**B**) Representative time-lapse images showing DiD-labeled SARS-CoV-2 binding to the pulmonary endothelium (arrows). Scale bars: 20 μm. (**C**) Representative time-lapse images showing DiD-labeled SARS-CoV-2 traveling (arrows) and interacting with platelets (arrowheads). Scale bars: 20 μm. (**D**) Representative time-lapse images starting at baseline. DiD-labeled SARS-CoV-2 is blue; platelets (anti-CD49b) are dark green; vasculature is labeled with nonspecific staining (light green). Top panels show platelet and SARS-CoV-2 signal; bottom panels show only SARS-CoV-2 signal. Circles highlight location of viral particles before or after anti–P selectin administration in control and treatment. Scale bars: 20 μm. (**E**) Quantification of blue (DiD-SARS-CoV-2) fluorescence intensity of the vasculature over time with or without addition of anti–P selectin antibody. (*N* = 3.) (**F**) Change in intensity measured at regions of interest containing viral particles, i.e., Δ after treatment (at 200 seconds) with anti–P selectin compared with baseline (at 0 seconds). Significance was determined by *t* test, **P < 0.01. (*N* = 3.) (**G**) One-phase decay trace showing fluorescence intensity (DiD-SARS-CoV-2) decay over time. Significance was determined by sum-of-squares *F* test. (*N* = 3.) (**H**) Rate of change over time with or without addition of anti–P selectin antibody. Significance was determined by 2-way ANOVA and Šidák’s test. (*N* = 3.) (**I**) Blinded quantification for the percentage of newly bound DiD-SARS-CoV-2 virus after treatment versus controls. Significance was determined by *t* test, *P < 0.05. (*N* = 3.) (**J**) Quantification of viral RNA after lethal inoculations with SARS-CoV-2 in anti–P selectin–treated mice versus control in lung. Significance was determined by *t* test, **P < 0.01. (*N* = 4.)

To study the physiological role of P selectin in SARS-CoV-2 trafficking, we visualized the movement of DiD-labeled SARS-CoV-2 clinical isolate in the infected lung in vivo. Before imaging, we confirmed that labeled authentic SARS-CoV-2 viral particles retained their expected tropisms for ACE2^+^ target cells by flow cytometry (Supplemental Figure 4, A and B). To monitor virus trafficking during SARS-CoV-2 infection, we first administered an intranasal dose of the SARS-CoV-2 isolate to transgenic mice expressing hACE2 (Figure 6A). Four days after infection, we injected fluorescently labeled virus intravenously and visualized viral distribution in lung vasculature using intravital pulmonary microscopy. Following intravenous injection, most labeled SARS-CoV-2 particles appeared immobile and bound to CD31^+^ endothelial capillary networks (Figure 6B). In some cases, we observed dynamic interactions between moving viral particles and (CD49b^+^) platelets, suggestive of transient binding events (Figure 6C). Individual particle tracking analyses revealed a mix of stationary and transient behaviors (Supplemental Figure 4C).

To directly assess the role of P selectin in these interactions, we injected animals with anti–P selectin and then evaluated changes in DiD-SARS-CoV-2 localization. Shortly after treatment, we observed a marked reduction in viral signal intensity within the lung microvasculature (Figure 6D and Supplemental Video 1). Quantification of fluorescence intensity showed a rapid decline in interaction of fluorescent SARS-CoV-2 particles with the endothelium following anti–P selectin antibody injection (Figure 6, E and F). Kinetic modeling of viral signal decay revealed a significantly faster clearance of viral particles in anti–P selectin–treated mice (decay constant *K* = 0.29) compared with controls (*K* = 0.002) (Figure 6G) and found that the change in viral clearance was maximum immediately after antibody injection (Figure 6H). Further, while control animals exhibited a steady-state accumulation of labeled SARS-CoV-2 particles, no new binding of viral particles was detected after animals received blocking P selectin antibody (Figure 6I).

Consistent with the intravital results showing that anti–P selectin prevented viral lodgment in the lung, we found lower viral RNA counts (Figure 6J) in the lungs of mice inoculated with SARS-CoV-2 with blocked P selectin (Figure 3G). This effect occurred independently of changes in major myeloid populations, including eosinophils, alveolar macrophages, inflammatory monocytes, or neutrophils (Supplemental Figure 5). Given the known susceptibility of hACE2-transgenic mouse models to SARS-CoV-2–associated neuroinvasion and encephalitis (29, 35–37), we next evaluated viral burden and immune cell profiles in brain tissue. Similarly to others, we found low levels of viral counts in the brain (Supplemental Figure 5A), but no statistical changes were seen across treatment groups. Similarly, myeloid populations remained unchanged (Supplemental Figure 5, B–G). However, anti–P selectin treatment during infection was associated with a shift in brain-resident T cell populations, characterized by increased CD4^+^ and decreased CD8^+^ T cells (Supplemental Figure 5, H and I). These data support that P selectin contributes to SARS-CoV-2 retention in the lung, and can shape downstream immune responses, including in the central nervous system. Overall, these results support a role for P selectin in modulating viral localization during SARS-CoV-2 infection in vivo.

## Discussion

Our team conducted a comprehensive functional analysis of the human genome to identify genes that when upregulated can suppress authentic SARS-CoV-2 infection. As a proof of principle, we focused on interactions between host anti–SARS-CoV-2 genes and the viral spike protein, and found that P selectin, a cell adhesion molecule expressed on activated platelets and endothelial cells, can bind to pathogenic coronavirus spike proteins and suppress infection; as an mRNA medicine this may have broad anti-coronavirus therapeutic utility. From a physiological perspective, we demonstrated that endogenous P selectin can also promote binding to SARS-CoV-2 spike, and these interactions may in part explain the coagulopathies that occur in COVID-19. Importantly, in vivo, P selectin in the lung controls the movement of viral particles within the vasculature, and overall, P selectin functions to protect the host from SARS-CoV-2–mediated disease.

SARS-CoV-2 infection in hACE2 AC70 mice results in rapid health deterioration, necessitating euthanasia at humane endpoints and thereby limiting survival analyses. Despite this, in vivo blockade of P selectin led to greater weight loss, indicating worsened clinical outcomes. Our intravital microscopy results showed viral clearance in the lung after blockade of P selectin, and similarly we confirmed reduced viral counts in infected mice treated with anti–P selectin antibodies. Immune profiling revealed no significant changes in pulmonary or cerebral myeloid populations; however, the brain exhibited increased CD4^+^ and decreased CD8^+^ T cell infiltration. This suggests a role for P selectin in regulating adaptive neuroimmune responses, particularly in sustaining CD8^+^ T cell–mediated antiviral activity and preventing dysregulated CD4^+^ T cell–driven inflammation, though further work is needed to establish whether there is a physiological implication of these effects.

From intravital imaging we observed viral particles interacting with the endothelium and platelets. These observations are congruent with ex vivo reports that SARS-CoV-2 particles accumulate and aggregate in vascular beds (38) spread through the body, or reports that platelets isolated from COVID-19 patients associate with SARS-CoV-2 RNA (39). At the cellular level, postmortem work on COVID-19 patients identified instances of endotheliitis and in some cases SARS-CoV-2 infection in endothelial cells (40). Although the level of endothelial infection in non-severe COVID-19 remains to be determined, it is possible that endothelial expression of P selectin could provide some protection from infection. We also show that surface P selectin can interact with SARS-CoV-1 and MERS spike proteins, suggesting that P selectin may play a broader role in protecting the host from coronavirus infection.

**Figure 7:**
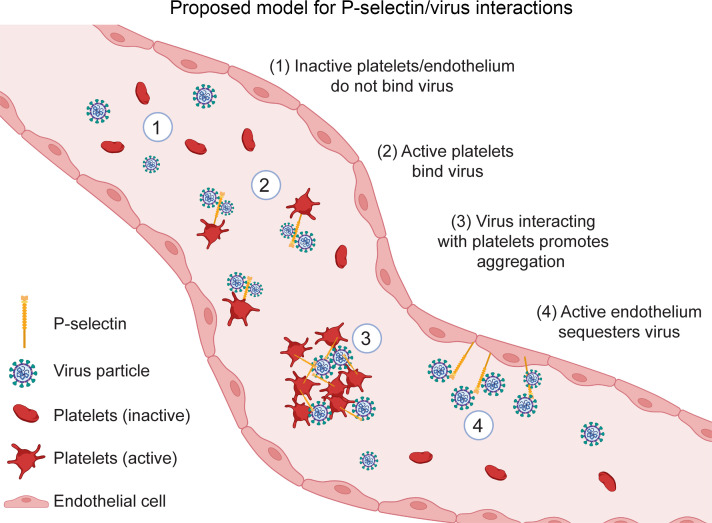
Proposed model for physiological P selectin function during SARS-CoV-2 infection. P selectin is mobilized to the plasma membrane of activated platelets and/or endothelial cells, where its surface expression influences infection outcomes. 1. In the absence of surface P selectin, resting platelets and endothelium do not bind viral particles. 2. Upon platelet activation, α-granules release P selectin to the surface, increasing viral particle binding. 3. This interaction promotes platelet aggregation and may increase prothrombotic events. 4. In endothelial cells, P selectin release from Weibel-Palade bodies enables sequestration of viral particles to the vascular walls, potentially altering viral dissemination and immune responses.

The physiological roles of P selectin in the context of SARS-CoV-2 infection are complex, and likely depend on disease stage, severity, and microenvironment. Multiple studies have investigated associations between P selectin expression and COVID-19, including correlations with disease severity. Generally most (34, 41–45) but not all studies (46–48) show that soluble P selectin expression increases with disease severity, and serum P selectin has shown sensitivity and specificity as a biomarker for long COVID (49). Interestingly, a recent clinical trial found no clinical effect of crizanlizumab, a blocking P selectin antibody, on the primary outcome of organ support–free days. However, in this large patient cohort study (422 patients), the authors also observed nearly double the number of hospital deaths (11.8% vs 6.7%) in the treatment group (50), although this effect was not found to be statistically significant. While P selectin/spike interactions may also promote clotting and drive pathology (see proposed model, Figure 7), this remains to be investigated in more detail; during acute SARS-CoV-2 infection P selectin appears to play a protective role in cells, mice, and patients.

Our study does have limitations. Other CRISPRa screens (51, 52) using human lung cell lines have highlighted additional modulators of SARS-CoV-2 pathology, for example mucins, that we did not detect. Similarly to P selectin, these membrane glycoproteins appear to reduce infection in *cis*. Overall the genes identified in a CRISPRa screen depend on the epigenetic state of the underlying cell line screened, and no one cell line accurately reflects the in vivo SARS-CoV-2 target cells. In vitro CRISPR screens provide insight into cell-autonomous mechanisms that can modify viral infection, and do not themselves fully recapitulate the mechanisms occurring during infection in vivo. However, when taken together these strategies can flag functionally relevant modifiers of SARS-CoV-2 infection that may have mechanistic or therapeutic value. For these reasons, it is important to mechanistically confirm CRISPR screening data via orthogonal and in vivo approaches as we have done here. For our in vivo work, ethically we could not evaluate survival directly. Moreover, the mouse model we used results in rapid death after infection. As such, we observe a small (albeit significant) effect from anti–P selectin injections. Further research using a mouse-adapted SARS-CoV-2 strain may help illuminate the role of P selectin during infection. In this case, as a proof of principle, we have confirmed a protective role of P selectin; however, multiple additional hits that we confirm in vitro remain to be confirmed in a more disease-relevant context. In the cell models we used to study P selectin function, we found statistically significant interactions with SARS-CoV-2 proteins; however, by FACS we observed both strong and weak spike-binding populations, indicating that other factors may modulate this interaction.

In summary, we report the unexpected finding that the leukocyte adhesion molecule P selectin binds to coronavirus spike proteins and plays a direct and protective role during SARS-CoV-2 infection.

## Methods

Further information can be found in Supplemental Methods.

### Sex as a biological variable.

Our study examined male and female animals, or human donor samples, and similar findings are reported for both sexes.

### Whole-genome CRISPR activation sgRNA lentivirus library preparation.

The human genome-wide CRISPRa-V2 library (Addgene 83978) was cotransfected with packaging plasmids pCAG-VSVG and psPAX2 (Addgene plasmids 35616 and 12260, respectively). Briefly, a T-175 flask of 80% confluent 293LTV cells (Cell Biolabs) was transfected in OptiMEM (Thermo Fisher Scientific) using 8 μg of the plasmid library, 4 μg pCAG-VSVG, 8 μg psPAX2, 2.5 μg pAdVantage (Promega), 30 μL of P3000 Reagent (Thermo Fisher Scientific), and 30 μL of Lipofectamine 3000 (Thermo Fisher Scientific). Cells were incubated overnight, and then medium was changed to Dulbecco’s modified Eagle medium (DMEM; Sigma-Aldrich) with 10% FBS and 1× GlutaMAX (Thermo Fisher Scientific). After 48 hours, viral supernatants were collected and centrifuged at 900 *g* for 10 minutes to get rid of cell debris. The supernatant was filtered through a 0.45 μm ultra-low-protein-binding filter (Merck Millipore). Aliquots were stored at –80°C.

### Whole-genome CRISPR activation screen.

HEK293-ACE2-SunTag cells were infected with the CRISPRa-V2 library (Addgene 83978) at a multiplicity of infection (MOI) of 0.3 (3 × 10^7^ cells, i.e., ~300 cells per guide) in T175 formats. Cells were selected with puromycin 1.6 μg/mL for 3 days. CRISPR-targeted cells were then inoculated with authentic SARS-CoV-2 (A.2.2) at a dose leading to 90% cell death after 72 hours (final MOI 0.02). Surviving cells were kept for an additional 72 hours with daily changes of medium. Additional control flasks were maintained throughout the same period without virus treatment. Cells were harvested for genomic DNA extraction. Screen was performed with 2 biological replicates per condition. Screen sequencing data are available online in the NCBI’s Gene Expression Omnibus database (GSE240991).

### Next-generation sequencing.

Genomic DNA corresponding to selected and diversity control samples were PCR-amplified with NEBNext High-Fidelity 2X PCR Master Mix (New England Biolabs) as described in ref. 27. The following primers were used in this reaction: NGSCRISPRiaF1 5′-CAGCACAAAAGGAAACTCACCCTAACTG-3′ and NGSCRISPRv2Rev1 5′-TGTGGGCGATGTGCGCTCTG-3′. A second PCR reaction with staggered primer mix P5 and P7 indexing primer unique to each sample was prepared as previously described (53). Reactions were isolated by gel electrophoresis and sent to the Ramaciotti Centre for Genomics (UNSW Sydney, New South Wales, Australia) for next-generation sequencing. Raw sequencing reads were mapped and counted using MAGeCK (v0.5.9.2) (54). Read counts were then analyzed using CRISPhieRmix (7) to identify enriched and depleted genes as compared with diversity controls. See Extended Supplemental Table 1 with gain-of-function and loss-of-function table for a full list of genes identified with CRISPhieRmix. The top 100 ranked genes, as determined by local FDR, were selected and uploaded to Ingenuity (v01-21-03) for core analysis (Ingenuity Pathway Analysis). KEGG pathway analysis was similarly performed on the top 100 ranked genes.

### Cell line generation for target validation using authentic SARS-CoV-2 virus.

SgRNA sequences for target genes were cloned into pLentiguide puro (Addgene 52963) and confirmed by Sanger sequencing. A full list of target sequences is found in Supplemental Table 3. Viruses were generated as described above. HEK-ACE2-SunTag clone used in the activation screen was infected with lentivirus sgRNAs and selected with puromycin for a period of 3 days before screening guides for target activation. RNA was extracted following the manufacturer’s protocol (Total RNA Kit, Favorgen). Supplemental Table 3 lists all sgRNA sequences used for CRISPRa. Cells were seeded as described in the *Authentic SARS-CoV-2 virus in vitro assays* section below.

### SARS-CoV-2 virus propagation.

Clade A.2.2 was isolated from GisAID hCoV-19/Australia/NSW2715/2020. Spike sequence was confirmed to be identical to the ancestral clade A first reported in Wuhan with only changes in the ORF making it unique, and classified as A.2.2. These studies used passage 3 virus, which is equivalent to less than 5 days in culture (initial swab was cultured for 3 days, and then a second passage was propagated for an additional 24 hours). This time in culture ensures the genome is the same as that reported in the original swab, as reported in ref. 55.

### Authentic SARS-CoV-2 virus in vitro assays.

SunTag CRISPRa cells were plated onto 384-well plates. Twenty-four hours later, cells were inoculated with serially diluted SARS-CoV-2 isolates in culture medium, where an equal volume was added to each well. For transient expression, tests were carried out in HEK-ACE2-TMPRSS2 cells generated as previously described (55). These cells were transfected as described in the *cDNA/mRNA transfection* section (Supplemental Methods) and seeded 24 hours after transfection. Cells were then inoculated as described above with isolates of SARS-CoV-2 variants (A.2.2 or Delta). Plates were incubated for 48 hours at 37°C before addition of NucBlue live nuclear dye (Invitrogen) at a final concentration of 2.5%. After a 4-hour incubation, plates were imaged using an IN Cell Analyzer HS2500 high-content microscopy system (Cytiva). Quantification of nuclei was performed with automated IN Carta Image Analysis Software and normalized to uninfected wells. Genes that did not reach statistical significance in the validation are reported in Supplemental Figure 1D.

### Cell line generation for spike binding screening.

SgRNA sequences for target genes were cloned into pXPR502 (Addgene 96923) and screened by Sanger sequencing. A full list of target sequences is found in Supplemental Table 1. Viruses were generated as described above, and transduced in a HEK cell clone expressing CRISPRa (synergistic activation mediator [SAM]) (Addgene 113341) previously described in ref. 27. Cells were incubated for a period of 3 days with 2 μg/mL of puromycin before testing of screening guides for SARS-CoV-2 spike binding capacity as described in *Flow cytometry — spike binding protein assays* (Supplemental Methods).

### Plaque assays.

VeroE6/TMPRSS2 cells were transfected with cDNA encoding GFP or SELP-GFP using Lipofectamine 3000. After 24 hours, cells were reseeded to reach 100% confluence on the next day. Inoculations were performed using stocks of SARS-CoV-2 A.2.2 passage 3 (Wuhan) that were serially diluted in DMEM (11995073, Gibco). This was done for a period of 60 minutes with gentle rocking every 15 minutes in the incubator. Plates were washed twice with PBS, and a solution containing 0.3% melted agarose in DMEM with 10% FBS was gently added to immobilize cell layers. Forty-eight hours after inoculation, cells were fixed with 4% paraformaldehyde (PFA), and agarose shields were removed before cell layers were stained for 5 minutes using 1% crystal violet, washed twice with distilled water, and allowed to dry. Plaque-forming units (PFU) were counted at the 10^–4^ dilution, at which distinct >10 plaques were best detected.

### SARS-CoV-2 spike labeling and conjugation.

The expression construct used to generate SARS-CoV-2 spike protein was a gift from Jason McLellan (Addgene 154754). Spike protein was prepared as described in ref. 27. Spike protein was conjugated to Alexa Fluor 647 using a protein labeling kit (catalog A20186, Invitrogen) according to the manufacturer’s instructions. Briefly, a solution of 50 μL of 1 M sodium and 500 μL of 2 mg/mL protein was prepared and incubated at room temperature for an hour. Conjugated protein was loaded onto a Bio-Rad BioGel P-30 Fine size exclusion purification resin column and eluted by centrifugation. Protein quantification was determined using NanoDrop (Thermo Fisher Scientific).

### Pseudotyped virus generation and DiD labeling.

Pseudotyped lentiviruses were generated as previously described (27). Briefly, pseudoviruses were generated using a 5-component plasmid system. SARS-CoV-2 (Wuhan) and Delta were made as previously described (27). Pseudotyped MERS and SARS-CoV-1 receptor protein sequences were cloned onto the same backbones as SARS-CoV-2 using NEBuilder HiFi DNA Assembly Master (New England Biolabs). DiD labeling was performed by adaptation of a previous protocol (56). Medium containing virus particles was first concentrated to 20× using 100,000-molecular-weight-cutoff protein concentrator columns (Thermo Fisher Scientific). Concentrated virus was then incubated using 1:2,000 DiD Cell-Labeling Solution (V22887, Thermo Fisher Scientific) for 20 minutes at 37°C. Medium was then diluted by a factor of 10, and reconcentrated using polyethylene glycol 8000 (PEG) overnight at 4°C. The next day the medium was centrifuged at 3,200*g* for 30 minutes at 4°C. The supernatant was discarded, and the pellet containing labeled viral particles was resuspended in DMEM and aliquoted. Virus was titered using the QuickTiter Lentivirus Titer Kit (Cell Biolabs Inc.) following the manufacturer’s conditions.

### DiD labeling of authentic SARS-CoV-2.

SARS-CoV-2 (Wuhan) isolates were expanded in VeroE6-TMPRSS2 cells as previously described (55). For labeling, medium containing virus isolates was incubated in DMEM (11995073, Gibco) with 1:10,000 Vybrant (V22887, Invitrogen) for 30 minutes at 37°C. This medium was resuspended with 1× PEG (ab102538, Abcam) and left overnight at 4°C. Medium was then centrifuged for 30 minutes at 3,200*g* at 4°C. The supernatant was removed, and the virus was resuspended, aliquoted, and stored at –80°C until use.

### Platelet isolation and activation.

Platelet isolation was performed as previously described (57, 58). Briefly, whole blood was centrifuged at 200*g* for 20 minutes. The supernatant (platelet-rich plasma) was collected and anti-coagulated further with citrate-dextrose. After incubating for 30 minutes at 37°C, the platelet-rich plasma was treated with 2 μM prostaglandin E_1_ (Sigma-Aldrich) and centrifuged at 800*g* for 10 minutes. The supernatant was discarded, and the platelet pellet was resuspended in HEPES-Tyrode’s buffer. Platelets were tested within 2 hours of isolation. Platelets were activated using thrombin (0.1 U/mL) (Sigma-Aldrich) for 7 minutes at room temperature in addition to fucoidan (100 nM), or, when relevant, blocking anti–P selectin antibody (10 μg/mL) (16, 59) or isotype control. CD62P-PE non-blocking antibody (12-0626-82, Thermo Fisher Scientific), spike–Alexa Fluor 647 (10 μg/mL), or DiD-Wuhan (3 × 10^7^ particles/mL) was then added for an additional 7 minutes. PPACK (60 nM) was then added to stop thrombin action. Platelets were then fixed in 4% PFA for 10 minutes before further analysis using flow cytometry. Additional antibody information is included on Supplemental Table 5. Platelet assays were performed from a minimum of 3 independent donors.

### Platelet aggregation assay.

Platelet aggregation was determined as previously reported (33). We incubated 2 × 10^6^ platelets for 5 minutes at room temperature with wheat germ agglutinin conjugated to either Alexa Fluor 555 or Alexa Fluor 647 (1:100). Labeled platelets were then recombined and incubated for 15 minutes with SARS-CoV-2 pseudovirus and corresponding doses of fucoidan (100 nM), anti–P selectin (20 μg/mL), or IgG control. Platelets were then fixed in 4% PFA for 10 minutes before analysis by flow cytometry. All assays were performed from a minimum of 3 independent donors.

### HUVEC gene editing.

HUVECs were obtained from umbilical cords by collagenase treatment. Cells were maintained in MesoEndo growth medium (Lonza) in a 5% CO_2_ incubator. HUVECs were cultured to 90% confluence. SELP-KO populations were generated using CRISPR lentivirus. Specifically, the vector lentiCRISPR v2^5^ was employed and packaged into viruses as described in Supplemental Methods. The sgRNA sequences 5′-GTCACAGATGAATTGACATG-3′ and 5′-ATAGTTCGGTGTGATAACTT-3′ were used to generate KO1 and KO2 populations, respectively. An intergenic control guide (5′- TGGCAACATATATAAGCAAG-3′) was used for control. Transduction was performed by an hour of centrifugation at 930*g* at 31°C in the presence of Polybrene (8 μg/mL). After spinfection, the medium was removed. Thirty-six hours after transduction, cells were treated with 0.5 μg/mL puromycin for 48 hours. Cells were then allowed to expand before subsequent assays. HUVEC characterizations were performed on a minimum of 3 independent donor cell lines. Figures displaying HUVEC KO and spike binding are normalized per donor sample, and statistical analyses were carried out using appropriate parametric tests as described in the legends.

### Microfluidics assay.

Microfluidic devices (57) were coated overnight at 4°C with fibronectin (100 μg/mL). The next day, 20,000 HEK cells transiently expressing GFP, ACE2-GFP, or SELP-GFP were loaded onto the inlet chamber in a volume of 10 μL and were allowed to settle for 3 hours. Perfusion with DiD-labeled pseudoviral particles was performed at a shear rate of 100 s^–1^ for a period of 1 hour per chip. All studies were completed within 4 hours after cells were settled. Chambers were washed with 100 μL of PBS, followed by 4% PFA fixation at room temperature for 20 minutes. Chambers were then washed with 100 μL of PBS and stained with Hoechst for 10 minutes, before a final wash with PBS. Slides were then imaged and quantified using Opera Phenix Plus (PerkinElmer). Microfluidic assays were repeated as 3 independent experiments.

### Flow cytometry of labeled authentic SARS-CoV-2.

Vero-E6-hACE2 and HeLa cells were lifted with TrypLE (Thermo Fisher Scientific) for 5 minutes at 37°C and neutralized with culture medium by centrifugation. One hundred thousand cells were resuspended in 20 μL of cell medium containing DiD-SARS-CoV-2 at 0.5 and 1 MOI or control medium. Cells were incubated for 45 minutes at 37°C. Cells were then fixed with 4% PFA for 30 minutes at room temperature, and washed twice with FACS buffer consisting of PBS, 2% BSA, 0.5 mM EDTA. Cells were then analyzed by flow cytometry using BD Canto.

### Mouse inoculations.

Transgenic mice expressing human ACE2 (hACE2) were purchased from Taconic Biosciences [B6;C3-Tg(CAG-ACE2)70Ctkt] and then bred in-house. All mice were housed in a specific pathogen–free, double-barrier unit at the University of Calgary. Mice were fed autoclaved rodent feed and water ad libitum. Eight- to twelve-week-old hACE2 mice were given 2.5 × 10^4^ PFU SARS-CoV-2 via the intranasal route. Briefly, mice were anesthetized under 3% isoflurane (with oxygen as carrier) for 2–3 minutes, and 25 μL of SARS-CoV-2 diluted in PBS was inoculated onto the nostril, until the mouse inhaled the drop. After 4 days, mice were anesthetized (10 mg/kg xylazine hydrochloride and 200 mg/kg ketamine hydrochloride), and the jugular was cannulated for the administration of labeled SARS-CoV-2 (4.8 × 10^6^ PFU). Three mice were used per condition. For infection course studies, mice were given 2.5 × 10^2^ PFU SARS-CoV-2 via the intranasal route and 50 or 100 μg/day i.p. of anti–P selectin blocking antibody (553741, BD Pharmingen) or control IgG (559157, BD Pharmingen).

### Flow cytometry for mouse tissues.

Cells were dissociated from lung or brain tissue as previously described (60, 61). Samples were captured using BD FACSCanto and analyzed using FlowJo software (BD Life Sciences). The following antibodies were used: rat anti–mouse CD45 (clone 30-F11, BioLegend), rat anti–mouse Ly6G (clone 1A8, BioLegend), anti–mouse CD11c (N418, eBioscience), rat anti–mouse Siglec-F (E50-2440, BD Biosciences), rat anti–mouse Ly6C (HK1.4, BioLegend), and rat anti–mouse CD11b (M1/70, eBioscience).

### Spinning disk confocal intravital microscopy.

Stabilized pulmonary intravital microscopy was performed as previously described (60). Briefly, anesthetized mice (ketamine and xylazine) received a right internal jugular intravenous catheter to administer fluorescent antibodies, DiD-SARS-CoV-2, and/or anti–P selectin treatment (100 μg; rat anti–mouse CD62P, BD Pharmingen). To visualize the endothelium and platelets, 5 μg of fluorescently conjugated anti-CD31 (clone 390, BioLegend; Alexa Fluor 594) and anti-CD49b antibodies (clone HMa2, BioLegend; Alexa Fluor 488), respectively, were administered intravenously 10 minutes before imaging. For traffic visualization of virus, DiD-SARS-CoV-2 (4.8 × 10^6^ PFU) was administered. The trachea of the mouse was exposed for the insertion of a small catheter, which was then attached to a ventilator (Harvard Apparatus). The mouse was placed in the right lateral decubitus position, and a small surgical incision was made. The intercostal muscles between ribs 4 and 5 were gently teased apart for the insertion of a lung window. The lung was then stabilized with a suction of 20 mmHg. Images were acquired with an upright microscope (BX51, Olympus) using a ×20/0.75 NA XLUM Plan F1 objective (Olympus). The microscope was equipped with a confocal light path (WaveFx, Quorum) based on a modified Yokogawa CSU-10 head (Yokogawa Electric Corp.). A 512 × 512–pixel back-thinned electron-multiplying charge-coupled device (EMCCD) camera (C9100-13, Hamamatsu) was used for fluorescence detection. At least 3 fields of view with identified foci corresponding to DiD-labeled SARS-CoV-2 were captured per animal. Images were acquired every ~13 seconds, for 10–20 minutes. Images were processed and analyzed in ImageJ2 v2.9.0/1.53t.

### Intravital quantification of DiD-SARS-CoV-2.

Video images were processed and analyzed in ImageJ2 v2.9.0/1.53t (Fiji) (62). Quantification was performed for the channel of interest by identification of foci using elliptical tools. Respective regions containing viral particles were measured for grayscale intensity throughout the recorded period and exported as a table. Rate of change (rolling average) in intensity was measured by calculation of the difference in intensity across 3 frames. Statistical analyses were performed with GraphPad Prism 9. For individual foci tracking, videos were blinded and manually tracked using the Manual Tracking plug-in, and individual particles were then centered and plotted using RStudio. Individual foci were also blindly evaluated to determine the number of new viral particles that were anchored throughout recordings.

### Statistics.

Statistical analyses were conducted using Prism 9.0 (GraphPad Software Inc.) unless indicated. *N* indicates the number of independent experiments. For animal studies or primary human cells (platelet or HUVEC experiments), *N* indicates the sample size. Data are represented as the mean ± SEM where applicable. When relevant, all statistical analyses were corrected for multiple comparisons. Additional information about statistical tests is reported in Methods and the figure legends.

### Study approval.

All experiments involving animals (Taconic Biosciences) were approved by the University of Calgary Animal Care Committee (protocol MO8131) and conformed to the guidelines established by the Canadian Council for Animal Care. HUVECs were sourced from consenting donors under ethics approval by the Sydney Local Health District Human Ethics Committee (X16-0225). For human platelet isolation, blood was obtained from healthy human donors in accordance with the Human Research Ethics Committee of the University of Sydney (2014/244) and the Declaration of Helsinki.

### Data availability.

Values underlying graphed data throughout this article are included in the Supporting Data Values file. Raw sequencing data can be accessed in the NCBI’s Gene Expression Omnibus under record GSE240991. Additional information may be requested from corresponding author GGN.

## Author contributions

CLM acquired and analyzed data (SARS-CoV-2 in vitro infections, pseudovirus data, and primary cell work), as well as performed in-depth/comprehensive analysis of live animal data, designed research studies, and wrote the manuscript. FVSC acquired and analyzed data (animal studies) and wrote the manuscript. AOS acquired and analyzed data (SARS-CoV-2 screen and validations) and wrote the manuscript. CLM, FVSC, and AOS share first authorship, and their listed order was determined by their overall contributions. FC, AA, AJC, LL, AD, YK, LH, JF, PC, RC, TYD, TI, MN, DH, SBC, JAC, and MPBM acquired and analyzed other datasets and participated in the experimental plans. JG, FP, ST, PK, and GGN provided project management, designed research studies, analyzed data, and wrote the manuscript.

## Funding support

This work is the result of NIH funding, in whole or in part, and is subject to the NIH Public Access Policy. Through acceptance of this federal funding, the NIH has been given a right to make the work publicly available in PubMed Central.

- National Health and Medical Research Council grants to GGN.
- Australian Research Council grants to GGN.
- Philanthropic donation from John and Anne Chong to GGN.
- Canadian Institutes of Health Research fellowship to FVSC.

## Supplementary Material

Supplemental data

Supplemental video 1

Supporting data values

## Version 1 11/17/2025

Electronic publication
