# Supplementary material for: P selectin promotes SARS-CoV-2 interactions with platelets and the endothelium
Source: J Clin Invest. 2025 Nov 17;135(22):e184514. doi: 10.1172/JCI184514 (PMC12618076; doi:10.1172/JCI184514)
Supplement: Supplemental data [file jci-135-184514-s056.pdf]

Supplemental

Methods

Lentivirus generation. Relevant vectors were co-transfected with packaging plasmids pCAG-VSVG and psPAX2 Addgene plasmids 35616 and 12260, respectively, using lipofectamine 3000 according to manufacturer's instructions. Molecular ratios of 3:3:1 corresponding to the vector of interest, psPAX2, and pCAG-VSVG were used to package viruses in HEK293FT cells. These cells were cultured in DMEM (Sigma-Aldrich) medium supplemented with 10% FBS, 1X GlutaMAX, 1X NEAA, and 1X PenStrep. Medium was replaced the day after transfection, and cells were cultured for an additional 24 hours. Medium was collected after this period and filtered through 0.45µm ultra-low protein binding filter (Merck Millipore) to remove cell debris. Medium was then concentrated using PEG (Abcam) according to manufacturer's instructions. Virus aliquots were stored at -80°C before use.

Generation of ACE2 and CRISPR-activation cell line. HEK293 cells (R70007, Invitrogen) expressing human ACE2 (previously described(27)) were transduced with lentiviruses carrying dCAS9-10xGCN4 (Addgene 60903) and pHRdSV40-scFv-GCN4-sfGFP-VP64-GB1-NLS (Addgene 60904) termed HEK293-ACE2-SunTag. Cells were then sorted and seeded as single clones on 96-well plates. Clones were screened for their ability to drive expression of a positive target gene by CRISPR-activation. Expression was determined by qRT-PCR (Supplemental Figure 1A).

RNA extraction and RT-qPCR. RNA was isolated from cells using the ISOLATE II RNA Mini Kit (Bioline) and concentration was measured by Nanodrop (Thermo Scientific). cDNA was synthesised using the iScript Select cDNA Synthesis Kit (Bio-Rad) according to manufacturer's

instructions. Briefly, 50-500 ng of RNA was added to iScript RT Supermix and nuclease-free water to a final volume of 10 µL. The assembled reactions were then incubated in a thermocycler as follows: 25°C for 5 min, 46°C for 20 min and then 95°C for 1 min. RT-qPCR was then performed on the cDNA samples using SYBR Select Master Mix (ThermoFisher Scientific) and the LightCycler 480 System (Roche). All primer sequences used are listed in Table S4. Results were analysed using the *ΔΔ*CT method and are presented in (Supplemental Figure 1E).

In vitro mRNA transcription. P-Selectin expression constructs were cloned into the Pfizer-BioNTech vector using NEBAssembly. mRNA was then generated by *in vitro* transcription, after linearisation using BspQI, and purification by ethanol precipitation. HiScribe® T7 mRNA Kit with CleanCap® Reagent AG (NEB, #E2080S) was used for mRNA production. mRNA samples were then aliquoted and stored at -80°C until used in expression and infection assays. P-selectin expression was confirmed by flow cytometry (Supplemental Figure 1I)

cDNA/mRNA transfection. A SELP-TurboGFP fusion construct was purchased from Origene (RG209822). TurboGFP control was generated by digesting SELP-GFP plasmid with MluI and BamHI, and ligating with annealed oligos (5'-CGCGCGAATTCATGCTAGCAACCGGTGCA-3', 5'-GATCTGCACCGGTTGCTAGCATGAATTCG-3'). The ACE2-GFP fusion construct was generated using NEBuilder HiFi DNA Assembly. Briefly this construct was made by PCR amplification of the SELP-GFP vector (5'- ACGCGTACGCGGCCG-3', 5'-GGCGATCGCGGCGGC-3'), and an ACE2-cDNA insert (5'-TGCCGCCGCGATCGCCATGTCAAGCTCTTCCTGGCTCC -3', 5'-GCGGCCGCGTACGCGTAAAGGAGGTCTGAACATCATCAGTGT – 3'). Cells were plated 24 hours before transfection on 6 well plates (720k per well). Cells were transfected with respective constructs (2.4 µg/well and 3.75ug/well, for cDNA and mRNA, respectively) using Lipofectamine

3000 according to manufacturer's protocols. Cells were replated for subsequent experiments after 48 hours of transfection.

Immunocytochemistry. Cells were fixed with 4% PFA for 20 minutes at room temperature. Cells were then blocked for an hour (PBS with 5% Normal Goat Serum, 1% BSA, 0.05% Triton-X, 0.3M Glycine). Cells were then incubated with respective antibodies (Table S3) in blocking solution for 1 hour at room temperature, followed by washes with PBS-0.05% Triton-X. Corresponding secondary conjugated antibodies and hoechst 33142 suspended in blocking solution was then used to incubate cells for an hour at room temperature. Cells were then washed with PBS-0.05% Triton-X, and PBS was added for imaging or placed on coverslip.

Flow Cytometry - Spike binding protein assays. Spike binding assays were performed as described in(27). Cells were dissociated with TryplE for 5 mins at 37C and then washed and then washed with culture media by centrifugation. A minimum of 100k cells were incubated with Alexa Fluor 647-conjugated SARS-CoV-2 spike glycoprotein (50ug/ml) for 30 mins at 4°C. Cells were then washed with 1% BSA in PBS, and resuspended in the same solution before analysis with the Cytek Aurora (Cytek Biosciences). 3 independent experiments were performed for these assays.

Flow Cytometry – Spike blocking antibody assays. Spike protein was blocked with clinical antibody batches as previously described in (25). Briefly, Alexa Fluor 647-conjugated SARS-CoV-2 spike glycoprotein (50ug/ml) was incubated for 1 hour at 37°C in PBS – 5% FBS solution with clinical antibody dilutions (1:2 series starting at 12.5 μg/mL for antibodies; corresponding concentrations of Igg-kappa were used for control). 150k HEK293 cells transfected with SELP-GFP (transfected for 48 hours) were then incubated with antibody/spike complex dilutions for 30

mins at 4°C. Cells were washed twice in PBS - 5%FBS and fixed in 4% PFA before proceeding to analyse by flow cytometry. GFP positive cells were gated, and fluorescent spike positive cells were recorded. Maximum spike binding was determined from samples stained with spike without antibody. Percentages displayed in figures are relative to the maximum spike bound. Clinical grade Sotrovimab (62.5 mg/mL; NDC 0173-0901-86) was kindly provided by GSK Healthcare while clinical grade Cilgavimab and Tixagevimab (100 mg/mL each; AstraZeneca) were kindly provided by Dr Sarah Sasson (Kirby Institute, UNSW). All flow cytometry data was analysed using FloJo Software v10.6 (BD Life Sciences).

High-throughput imaging. Cells were imaged using a Perkin Elmer's Opera Phenix. High-throughput image analysis was performed using Harmony Software (Perkin Elmer). Imaging analysis and quantification for microfluidic studies and lentiviral work was performed using Harmony High-Content Imaging and Analysis Software (Perkin Elmer).

Alphafold 3 structural prediction. The full-length sequence for SARS-CoV-2 spike and P-Selectin were submitted to AlphaFold3 with default settings and parameters (24). The output structure was visualised using ChimeraX (63). For visualisation of interface, non-interacting protein domains were excluded for depiction.

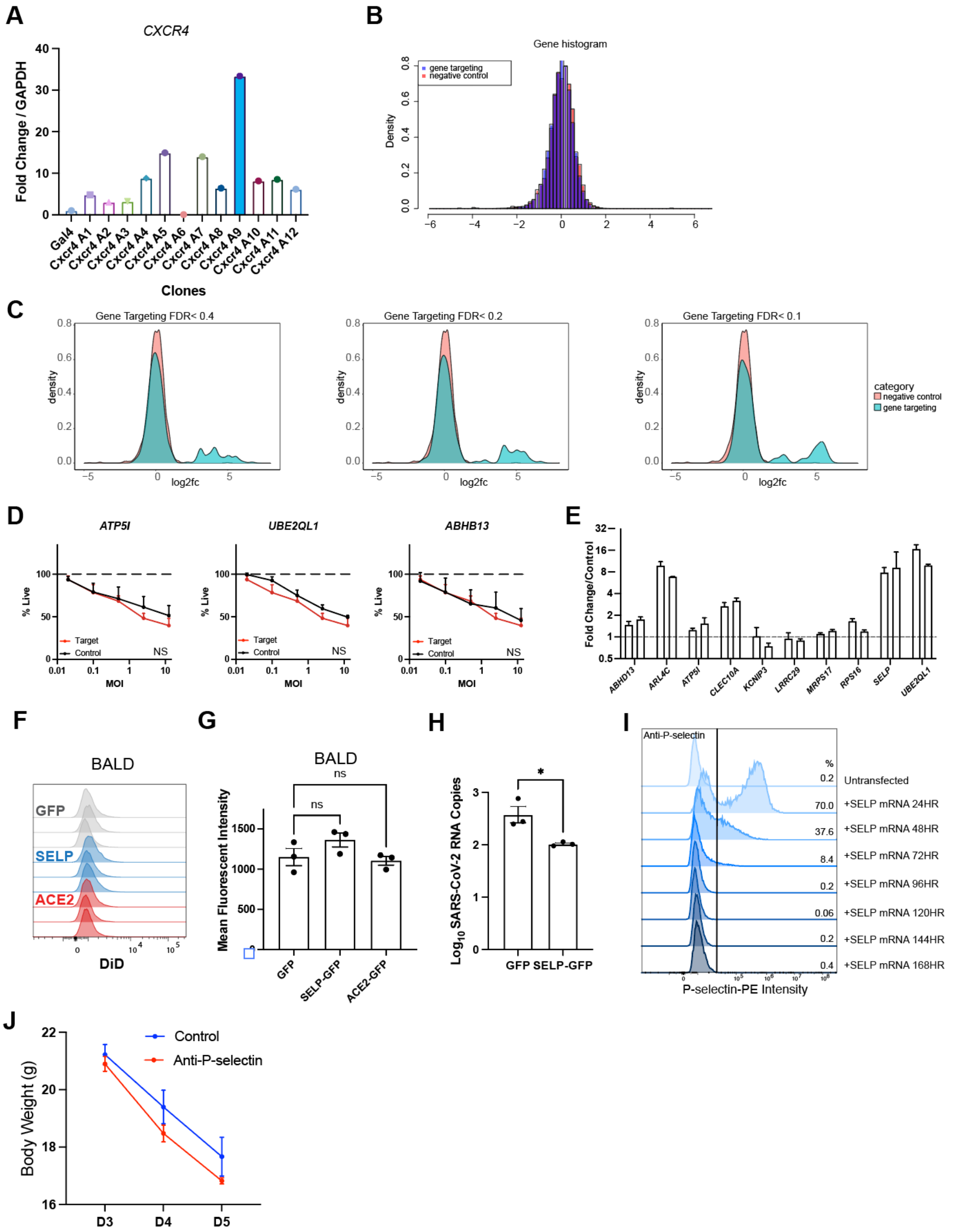

Figure 1. Additional data from activation screen and spike assays. (A) qRT-PCR screen showing expression of *CXCR4* by dCAS9-Suntag machinery in HEK-ACE2 cells. Blue bar indicates clone selected for future studies. (B) Superimposed histograms displaying negative control guides with targeting genes showing normal distributions. (C) Density diagrams showing

superimposed negative distribution vs. gene targets with decreasing False Discovery Rates (FDR). (D) Individual target validation that did not show significant protection against SARS-CoV-2. (E) Expression of target genes by each individual sgRNA and normalized to control negative guides. (F) Flow cytometry histograms and (G) quantification of binding intensity presented for bald pseudovirus. Significance was determined by 1-way ANOVA and Dunnett test. (H) Virus production quantified by RT-qPCR from media of GFP or SELP-GFP expressing cells infected with SARS-CoV-2 A2.2 (MOI 0.02 48 hrs). Significance was determined using t-test, $P<0.05$ (N=3). (I) Flow cytometry histogram of P-selectin mRNA transfected cells showing timecourse of P-selectin-PE antibody staining. (J) Body weights recorded throughout infection with authentic SARS-CoV-2 administered Anti-P-selectin or control antibodies.

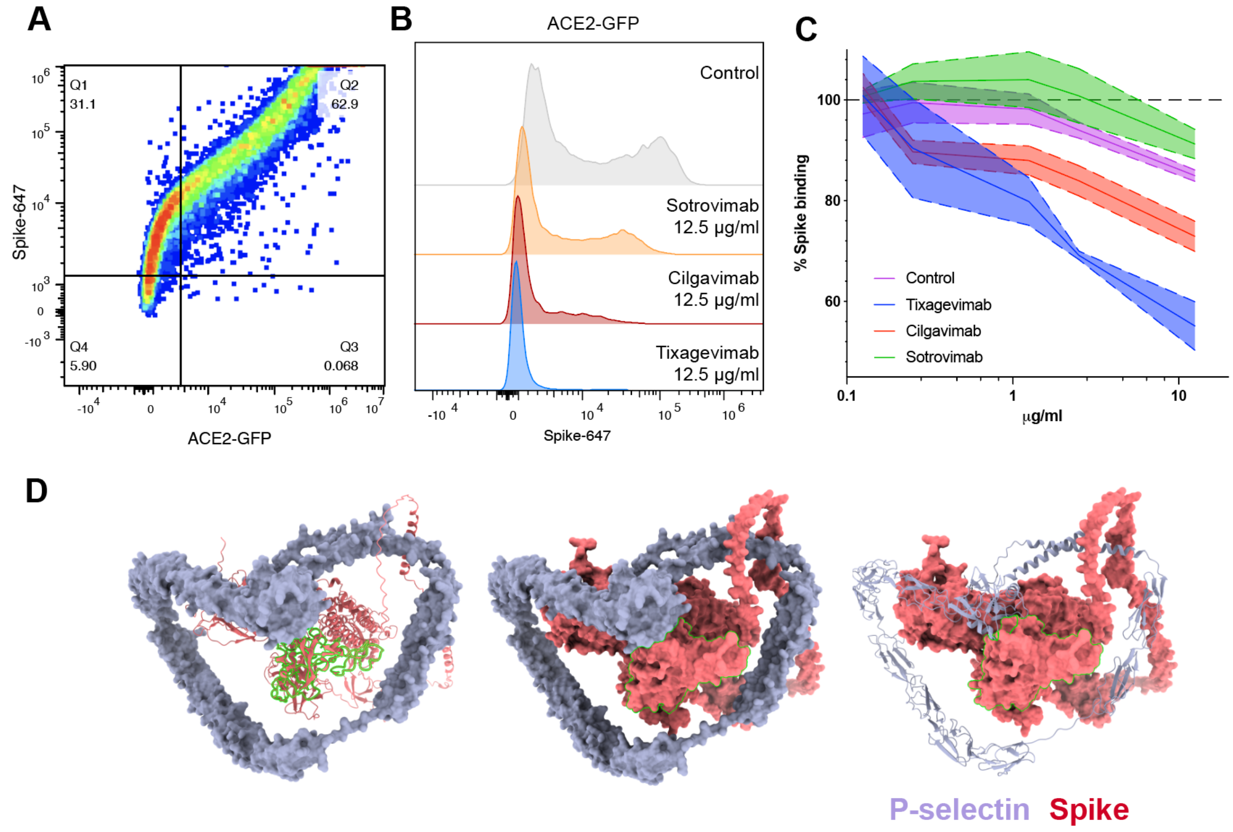

Figure 2. Spike binding validation and P-selectin/Spike blocking assays. (A) Sample flow cytometry plot showing ACE2-GFP expressing cells binding SARS-CoV-2 Spike-647. (B) Flow cytometry histograms showing binding of antibody-blocked spike protein in HEK293T-ACE2-GFP expressing cells. (C) Summary of dose-response curves showing binding of antibody-blocked spike protein in HEK293T-SELP-GFP expressing cells. (D) Alphafold3 model of complete P-selectin/Spike proteins. RBD is highlighted in green.

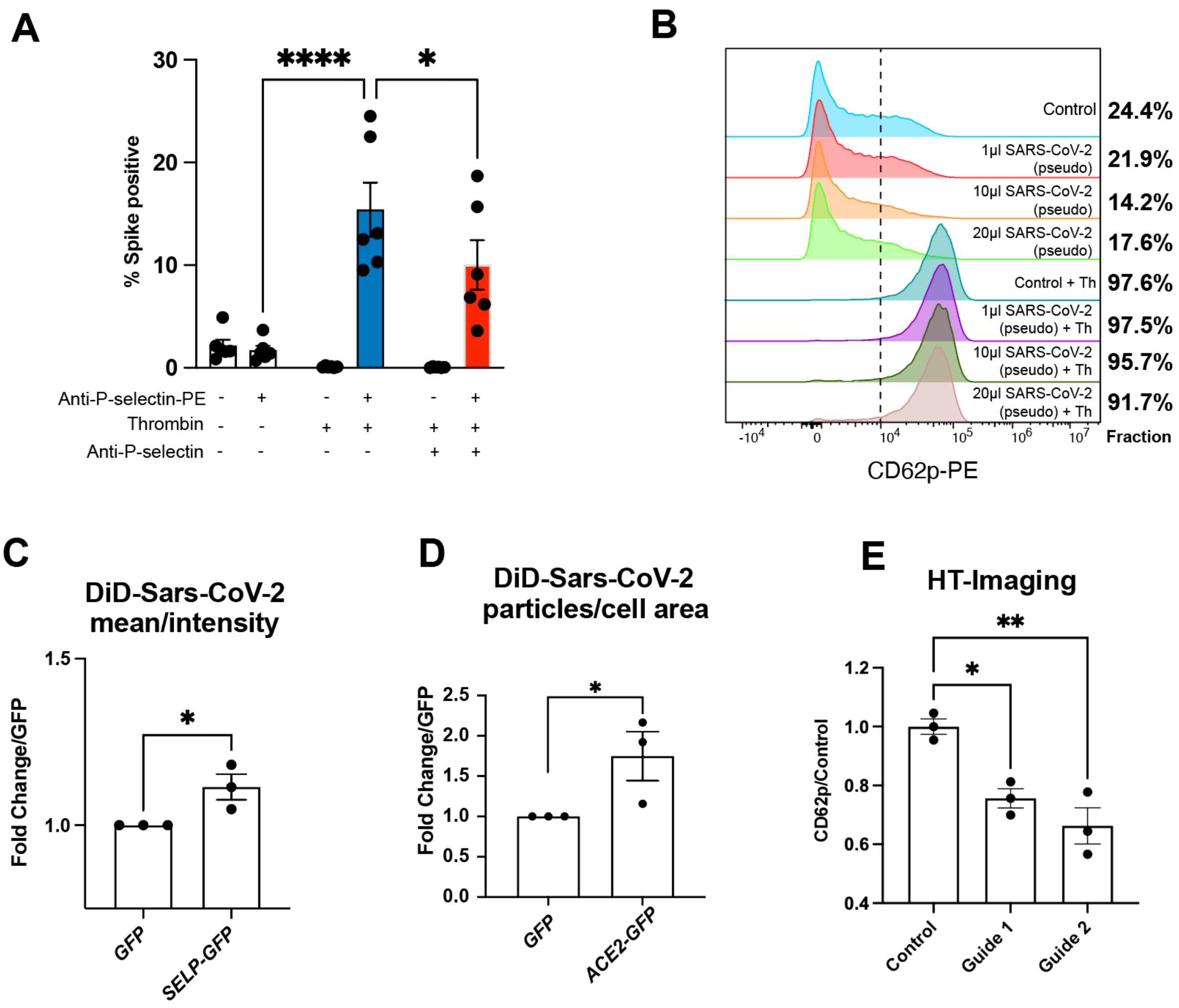

Figure 3. Spike and DiD-Sars-CoV-2 binding assays. (A) Quantification of spike fraction found in P-selectin positive platelets. Significance was determined by 2-way ANOVA and Sydak test, *P<0.5. (B) P-selectin expression in control vs. low thrombin (0.02 U/mL) treated platelets exposed to SARS-CoV-2 Pseudovirus. (C) Quantification of DiD-pseudovirus fluorescent intensity in microfluidic capillaries lined with SELP-GFP cells or controls. (D) Quantification of DiD-pseudovirus fluorescent particles in microfluidic capillaries lines with ACE2-GFP or controls. Significance was determined by t test *,P<0.05. (E) High-throughput imaging quantification of CD62p positive HUVECs after CRISPR knock out of SELP. Significance was determined by 1-Way ANOVA and Dunnetts, **, P<0.01; *, P<0.05.

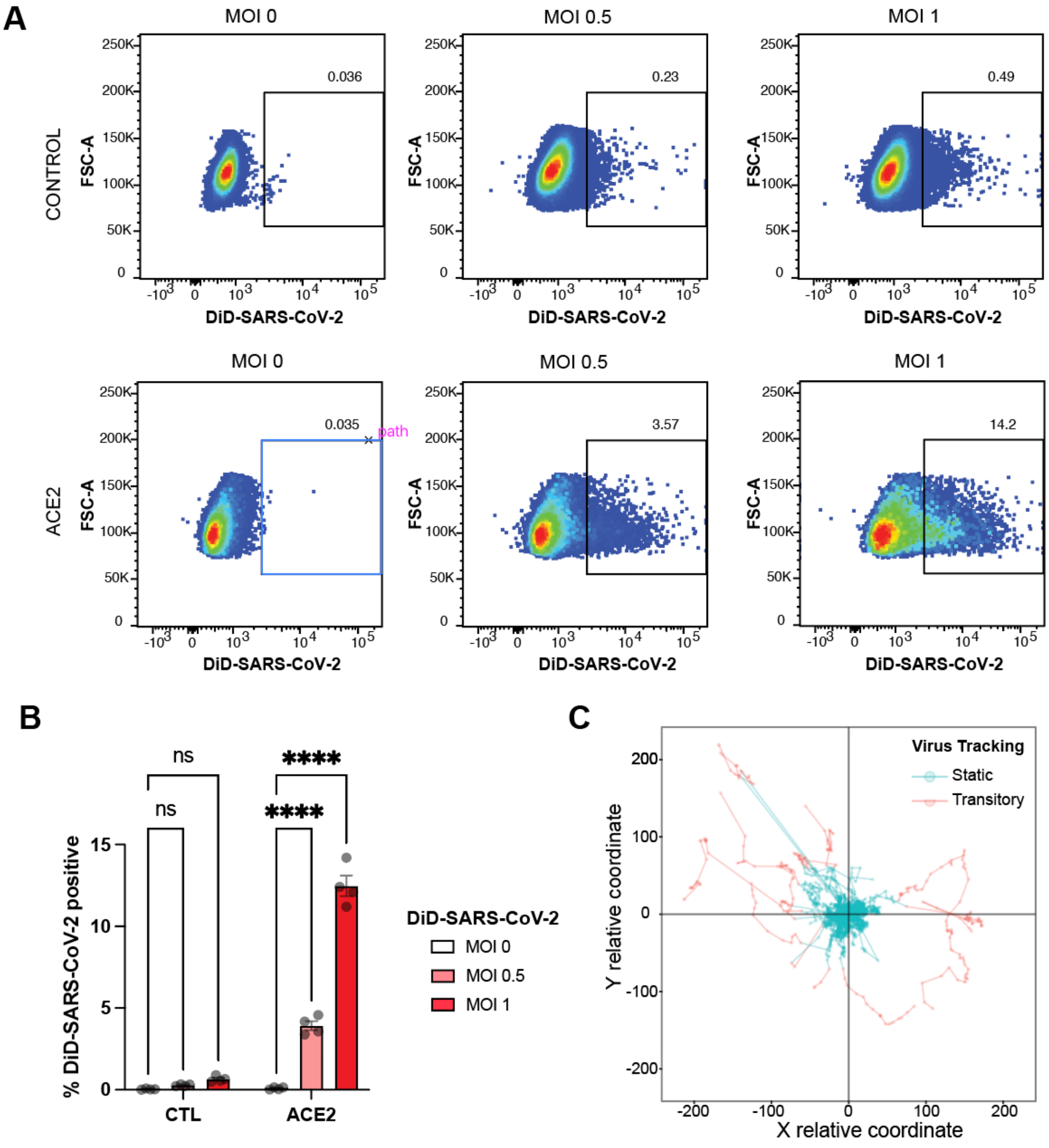

Figure 4. Authentic DiD-SARS-CoV-2 binding validation and *in vivo* tracking. (A) Sample flow-cytometry plot showing preferential binding of DiD-SARS-CoV-2 in control cells or Vero6-hACE2 cells. (B) Percent of DiD-SARS-CoV-2 cells. Significance was determined by 2-Way ANOVA and Dunnets ****, P<.0001. (C) Individual tracks of DiD-labelled particles, compared from a central origin point.

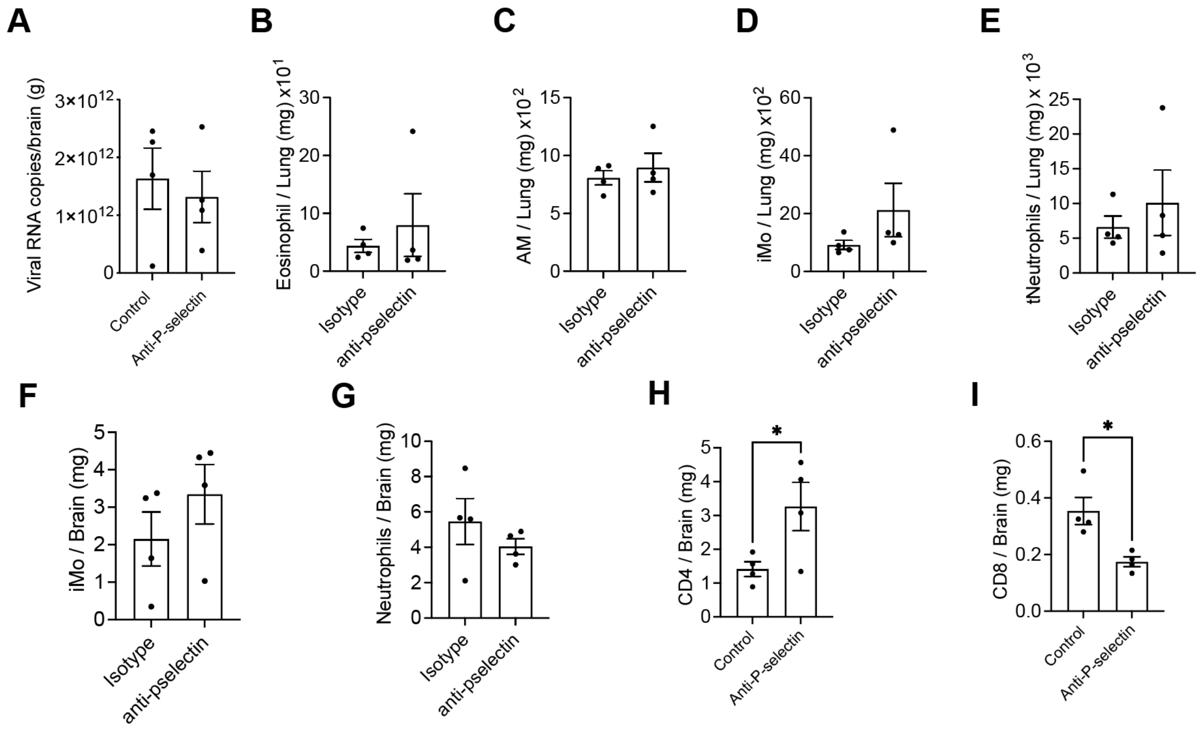

Figure 5. Additional immune profiles of lung and brain tissues of SARS-CoV-2 infected mice. (A) Viral counts as determined by qRT-PCR in the brain of infected mice treated with Anti-P-selectin or isotype control. Quantified (B) Eosinophils, (C) alveolar macrophages, (D) inflammatory monocytes and (E) neutrophils in lung samples of infected mice treated with Anti-P-selectin or isotype control. Quantified (F) inflammatory monocytes and (G) neutrophils in the brain of infected mice treated with Anti-P-selectin or isotype control. Quantification of CD4+ (H) and CD8+ T-cells (I) in the brain. Significance was determined by t-test *, P<.05. (N=4)

Table 1. CRISPR-Activation screen for overrepresented genes with FDR <0.1.

Gene	locFDR	Score	Entrez Gene Name	Location	Type(s)
UBE2QL1	0.0399761	0.96002393	ubiquitin conjugating enzyme E2 Q family like 1	Nucleus	enzyme
SELP	0.0493636	0.95063639	selectin P	Plasma Membrane	transmembrane receptor
ARL4C	0.0504077	0.94959227	ADP ribosylation factor like GTPase 4C	Nucleus	enzyme
RPS16	0.0534214	0.94657861	ribosomal protein S16	Cytoplasm	other
MRPS7	0.0553742	0.94462578	mitochondrial ribosomal protein S7	Cytoplasm	other
ABHD13	0.0555443	0.94445567	abhydrolase domain containing 13	Other	peptidase
LRRC29	0.0561946	0.94380536	leucine rich repeat containing 29	Other	enzyme
ATP5I	0.0582609	0.94173907	ATP synthase membrane subunit e	Cytoplasm	transporter
KCNIP3	0.0591365	0.94086349	potassium voltage-gated channel interacting protein 3	Nucleus	transcription regulator
CLEC10A	0.0592463	0.94075369	C-type lectin domain containing 10A	Plasma Membrane	other
SENP7	0.0592691	0.9407309	SUMO specific peptidase 7	Nucleus	peptidase
SYNRG	0.0604974	0.93950264	synergin gamma	Cytoplasm	other
STX2	0.0703712	0.92962882	syntaxin 2	Cytoplasm	transporter
RPA2	0.0705743	0.9294257	replication protein A2	Nucleus	other
TAOK3	0.0788244	0.9211756	TAO kinase 3	Cytoplasm	kinase
CAPN11	0.0866859	0.91331413	calpain 11	Cytoplasm	peptidase
ASB11	0.0899016	0.91009843	ankyrin repeat and SOCS box containing 11	Nucleus	transcription regulator

DNAJC8	0.0958588	0.90414118	DnaJ heat shock protein family (Hsp40) member C8	Nucleus	other
NRIP1	0.0960719	0.90392811	nuclear receptor interacting protein 1	Nucleus	transcription regulator
ETV2	0.0960901	0.90390989	ETS variant transcription factor 2	Nucleus	transcription regulator
RAB12	0.0961285	0.90387155	RAB12, member RAS oncogene family	Cytoplasm	enzyme
TRIM58	0.0962703	0.90372971	tripartite motif containing 58	Other	enzyme
LANCL3	0.0992948	0.90070519	LanC like 3	Other	other

Table 2. CRISPR-Activation screen for top 10 underrepresented genes.

**Gene**	**locFDR**	**Score**	**FDR**
ATP6AP1L	0.30500658	0.69499342	0.30500658
GFM2	0.48960427	0.51039573	0.39730543
PEPD	0.69577662	0.30422338	0.49679582
PICK1	0.69745181	0.30254819	0.54695982
COX7C	0.71498018	0.28501983	0.58056389
GPKOW	0.71777868	0.28222132	0.60343302
MMP3	0.72229919	0.27770082	0.6204139
KLK1	0.72337579	0.27662422	0.63328414
UTP18	0.7317411	0.2682589	0.6442238

Table 3. CRISPR-Activation sgRNA oligos cloned for individual target evaluation.

Target gene	Forward Oligo	Reverse Oligo
CLEC10A_SG1	caccGCTCCCAGATGGTAAAATG	aaacCATTTTACCATCTGGGAGC
CLEC10A_SG2	caccGGAAGCTCCGAGAAGAGGAA	aaacTTCCTCTTCTCGGAGCTTCC
CLEC10A_SG3	caccgAATGAGGAAGCTCCGAGAAG	aaacCTTCTCGGAGCTTCCTCATTCc
SELP_SG1	caccgTGATAATGCAAGAGTAATGT	aaacACATTACTCTTGCATTATCACc
SELP_SG2	caccgTAGAAATAGTGGTTGCCAAG	aaacCTTGGCAACCACTATTTCTACc
SELP_SG3	caccgTATCAACATTCTAACTTCAT	aaacATGAAGTTAGAATGTTGATACc
ARL4C_SG1	caccGGACCGCGCCTGACCCCCGG	aaacCCGGGGGTCAGGCGCGGTCC
ARL4C_SG2	caccgCGCGAAAACGGCCGGCTGGG	aaacCCCAGCCGGCCGTTTTCGCGCc
ARL4C_SG3	caccGCGCGCGAAAACGGCCGGCT	aaacAGCCGGCCGTTTTCGCGCGC
RPS16_SG1	caccgCTTCTGTTGCGGCTAACGGG	aaacCCCGTTAGCCGCAACAGAAGCc
RPS16_SG2	caccgAGGCCCTTAGGCGCTCAGAG	aaacCTCTGAGCGCCTAAGGGCCTCc
RPS16_SG3	caccGAGCTTTACGGCTTCTGTTG	aaacCAACAGAAGCCGTAAAGCTC
MRPS7_SG1	caccgAACTCTAACGTCCAGATCAG	aaacCTGATCTGGACGTTAGAGTTCc
MRPS7_SG2	caccgTTTCACTCCGGGGTCCAAAG	aaacCTTTGGACCCCGGAGTGAAACc
MRPS7_SG3	caccgTGCGTTTCTCTCCACTGATC	aaacGATCAGTGGAGAGAAACGCACc

ABHD13_SG1	caccgTCTGCGCATGCTCGGAAGAG	aaacCTCTTCCGAGCATGCGCAGACc
ABHD13_SG2	caccgCTGCCAGTGAGCCCCCGCGA	aaacTCGCGGGGGCTCACTGGCAGCc
ABHD13_SG3	caccgCCAGTGAGCCCCCGCGACGG	aaacCCGTCGCGGGGGCTCACTGGCc
LRRC29_SG1	caccGCAATCACTAAGCACCACAT	aaacATGTGGTGCTTAGTGATTGC
LRRC29_SG2	caccGATGTGGTGCTTAGTGATTG	aaacCAATCACTAAGCACCACATC
LRRC29_SG3	caccGTCGCAAAAGCTGACGACTT	aaacAAGTCGTCAGCTTTTGCGAC
ATP5I_SG1	caccgAGTCACCCTTCGGTCGCCAG	aaacCTGGCGACCGAAGGGTGACTCc
ATP5I_SG2	caccgCAGTCACCCTTCGGTCGCCA	aaacTGGCGACCGAAGGGTGACTGCc
ATP5I_SG3	caccGTCACCCTTCGGTCGCCAGG	aaacCCTGGCGACCGAAGGGTGAC
KCNIP3_SG1	caccGGAGGGAGCGAGGAGGCAGA	aaacTCTGCCTCCTCGCTCCCTCC
KCNIP3_SG2	caccGAGGGAGCGAGGAGGCAGAG	aaacCTCTGCCTCCTCGCTCCCTC
KCNIP3_SG3	caccGGAGCGAGGAGGCAGAGGGG	aaacCCCCTCTGCCTCCTCGCTCC
NEG1	CACCGCTGCACTGTGATAACAATAG	aaacCTATTGTTATCACAGTGCAG
NEG2	CACCGCATTCTTCAAACTATCCCA	aaacTGGGATAGTTTGAAGAATG

Table 4. Primers used in RT-qPCR.

Target gene	Forward Primer	Reverse Primer
CLEC10A	AGCAACTTCACCTCAAACACTG	AGATGCTATCGTTTCTTCCAAGC
UBE2QL1	CCTGTTCGACTGGAACGTGAA	GATGAACTCGGTGTTGGTCTC
SELP	ACTGCCAGAATCGCTACACAG	CACCCATGTCCATGTCTTATTGT
ARL4C	CCAGTCCCTGCATATCGTCAT	TTCACGAACTCGTTGAACTTGA
RPS16	TCGGACGCAAGAAGACAGC	AGCAGCTTGTACTGTAGCGTG
MRPS7	GAGGAGAAATATGTTCGGGAGC	CGTTCGATGGTTGCCTGTT
ABHD13	GATACACTGGAGACAATTCACCC	ACCTGTGACCTATGTTGCCTG
LRRC29	TGCCAGAACTCACAGACAACG	CTGGACAGGTTGAGATGCTGC
ATP5I	CAGGTCTCTCCGCTCATCAAG	GCCCGAGGTTTTAGGTAATTGT
KCNIP3	CAGTCTCTCTACAGGGGCTTT	AGAACTGCGCGTAAATGAGTTT

Table 5. Antibodies.

Name	Catalog/Clone	Company
CD62p-PE	12-0626-82/Psel.KO2.3	Thermofischer Scientific
CD62p	14-0628-82/AK-4	Thermofischer Scientific
CD62 Blocking antibody	G1	Made in house from hybridoma.
ACE2-AlexaFluor488	SANTSC-390851 AF488/ E11	Santa Cruz
ACE2	MA5-32307/SN0754	Thermofischer Scientific
SARS-CoV-2 Spike Protein S1	PA5114528/polyclonal	Thermofischer Scientific
Sotrovimab	S309	GSK Healthcare
Cilgavimab	AZD1061	Astrazeneca
Tixagevimab	AZD8895	Astrazeneca

*R.P. McEver, Oklahoma Medical Research Foundation, was obtained from the Developmental Studies Hybridoma Bank, created by the *Eunice Kennedy Shriver* National Institute of Child Health and Human Development of the National Institutes of Health and maintained at The University of Iowa, Department of Biology, Iowa City, IA 52242.
